# Exploring the Big Data Paradox for various estimands using vaccination data from the global COVID-19 Trends and Impact Survey (CTIS)

**DOI:** 10.1126/sciadv.adj0266

**Published:** 2024-05-31

**Authors:** Youqi Yang, Walter Dempsey, Peisong Han, Yashwant Deshmukh, Sylvia Richardson, Brian Tom, Bhramar Mukherjee

**Affiliations:** ^1^ Department of Biostatistics, University of Michigan, Ann Arbor, MI, USA.; ^2^ Biostatistics Innovation Group, Gilead Sciences, Foster City, CA, USA.; ^3^ Center For Voting Opinions and Trends in Election Research, Noida, India.; ^4^ MRC Biostatistics Unit, University of Cambridge, Cambridge, UK.

## Abstract

Selection bias poses a substantial challenge to valid statistical inference in nonprobability samples. This study compared estimates of the first-dose COVID-19 vaccination rates among Indian adults in 2021 from a large nonprobability sample, the COVID-19 Trends and Impact Survey (CTIS), and a small probability survey, the Center for Voting Options and Trends in Election Research (CVoter), against national benchmark data from the COVID Vaccine Intelligence Network. Notably, CTIS exhibits a larger estimation error on average (0.37) compared to CVoter (0.14). Additionally, we explored the accuracy (regarding mean squared error) of CTIS in estimating successive differences (over time) and subgroup differences (for females versus males) in mean vaccine uptakes. Compared to the overall vaccination rates, targeting these alternative estimands comparing differences or relative differences in two means increased the effective sample size. These results suggest that the Big Data Paradox can manifest in countries beyond the United States and may not apply equally to every estimand of interest.

## INTRODUCTION

The use of internet-based surveys has become increasingly common in population-based research. These surveys involve electronically sending a set of questions to participants and can be distributed via email, social media accounts, or as a pop-up survey when visiting a website (*1*). Survey researchers can administer questionnaires through internet-based platforms at a relatively low cost to a potentially large number of respondents (*2*). These surveys often result in a nonprobability sample. A nonprobability sample may lack a well-defined source population or sampling frame. Even if a sampling frame can be defined, the probability of selecting a specific sample from the target population remains unknown (*3*). Nonprobability sample surveys may suffer from selection bias, which can lead to invalid inference (*4*). In a total survey error framework, one such source of the bias is coverage bias, as respondents to online questionnaires are limited to those with access to the internet (*5*). The distinction between the sampling frame and the target population can affect the generalizability and external validity of any statistical inference procedure. Nonresponse bias is another source of bias that arises when respondents and nonrespondents differ significantly in the outcome of interest (*6*). Online surveys have reportedly low response rates (*7*), though they can achieve large sample sizes by reaching a much larger pool of potential participants. In the absence of a standard probability survey design, respondents are self-selected, which can negatively affect representativeness (*2*). For instance, those who are more concerned about severe acute respiratory syndrome coronavirus 2 infection may be more likely to respond to a COVID-19–related questionnaire (*8*). With a large sample size resulting in very small variance for the resultant estimates, and biases that do not diminish with sample size, the squared bias term dominates the mean squared error (MSE) of the estimator. Thus, for a big self-selected sample as obtained from an internet-based survey, the focus of our statistical thinking should be on controlling bias, rather than minimizing variance (*9*).

For estimating COVID-19 vaccine uptake, the official population-level data collected by government agencies are often publicly available as a reliable benchmark (*10*–*12*). The existence of such gold standard data gives researchers the opportunity to quantify estimation error, defined as the difference between the sample estimate from survey data and the true population value of vaccine uptake rates. For example, using reports from the U.S. Centers for Disease Control and Prevention (CDC) as the benchmark, researchers found that the COVID-19 Trends and Impact Survey (CTIS), which is a large nonprobability sample administered through Facebook/Meta with a weekly average sample size of around 250,000, produced a more biased estimate of the first-dose vaccination rate among U.S. adults when compared to a small probability survey (Axios-Ipsos) of an average sample size of around 1000 per wave (*13*). The study by Bradley *et al.* (*13*) used a decomposition framework proposed by Meng (*9*), describing what is now known as the “Big Data Paradox.” The main result provides an expression for the difference between the sample mean and the population mean (defined as estimation error) into a product of three terms, which are described as follows (*9*)

$$\begin{array}{c} \text{Estimation}\;\text{error}=\text{Data}\;\text{defect}\;\text{correlation}\;\times \;\text{Data}\;\text{deficiency}\;\times \\ \text{Inherent}\;\text{problem}\;\text{difficulty} \end{array}$$

The first term, the data defect correlation (ddc), quantifies the extent of selection bias by measuring the correlation between the value of the response and the reporting/recording indicator. The second term, data deficiency, is related to the amount of sample data collected. The third term, inherent problem difficulty, is the population SD of the response. This decomposition framework shows that even with vast amounts of data, a small ddc can induce a huge estimation error when compared to a simple random sample (SRS) of a much smaller size. On the basis of the study by Bradley *et al.* (*13*), one may naturally ask the question of whether surveys like CTIS that were carried out globally through 2 years of the pandemic have any substantive value, particularly for low- and middle-income countries (LMICs) where limited disaggregated data are publicly available for research (*14*).

Large online surveys like the CTIS, which gather individual-level data on COVID-19 vaccination, can provide valuable insights beyond the overall vaccination rate. These include the variation of vaccination rates across socio-demographic factors and health-related behaviors, which may not be captured by the aggregate data released by the official national statistics (*15*–*17*). They may also be able to capture changes over time as a result of policy interventions. The more granular level data from surveys can help identify vulnerable subgroups with less access to the vaccine and less trust in the health care system (*18*–*21*). This may lead to targeted intervention and campaign strategies. Thus, despite the reality of the Big Data Paradox for estimating the overall vaccination rates, or more broadly, overall prevalence rates, these nonprobability samples have been applied widely to reveal relative/comparative patterns (*22*).

**Fig. 1.**
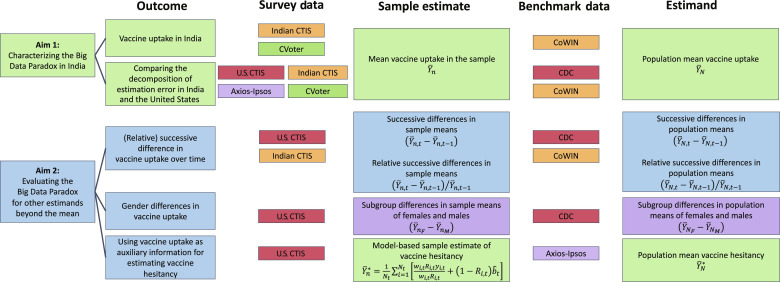
Study objectives and methodology. Our study is driven by two primary objectives. First, we assess the applicability of the Big Data Paradox to India. We also conduct a comparative analysis with the United States concerning the three components of estimation error. Second, we explore the potential of biased survey data to yield more accurate estimates through the modification of the estimand. This exploration involves an examination of accuracy across new estimands, including successive and subgroup differences, as well as model-based estimates.

This paper has two main objectives (Fig. 1). First, as a parallel to the study by Bradley *et al.* (*13*), we aim to evaluate the accuracy of the CTIS in estimating the first-dose COVID-19 vaccination rate in the Indian adult population. Our goal is to investigate whether the Big Data Paradox that has been explored in the United States also holds for a populous country like India with a completely different fabric of society and health care system. In addition to CTIS, we used a population-based sample survey conducted via computer-assisted telephone interviewing (CATI) in India during the contemporary time frame. We compared both of these COVID-tracker surveys to the gold standard official data released by the Government of India. Second, we sought to determine whether a survey that is substantially biased in estimating the population average of the target variable could still be useful if the estimand is changed to (i) the successive difference or relative successive difference in the population average over time; (ii) differences in population averages of two subgroups (such as gender); and (iii) the population average of a correlated variable (such as vaccine hesitancy) for which gold standard population-level official statistics may not be available. Because the U.S. CTIS produced a biased estimate of the population-level first-dose COVID-19 vaccination rate (*13*), we studied the above three problems using the U.S. CTIS data. We also investigated the estimand (i) using the Indian CTIS data, while (ii) and (iii) were not explored in India due to the limited availability of disaggregated benchmark data for vaccine uptake and probability surveys on vaccine hesitancy, respectively.

**Fig. 2.**
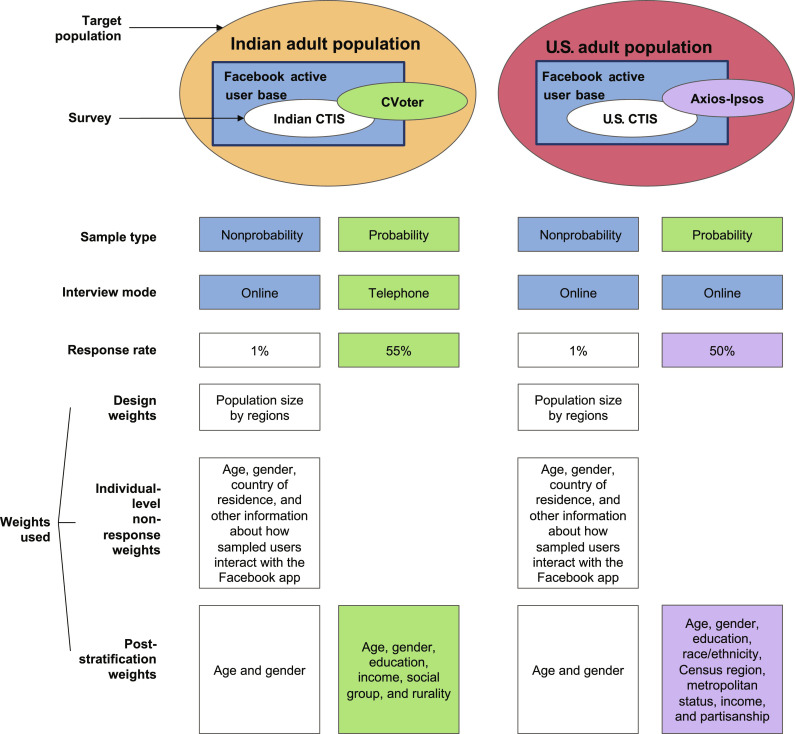
Data sources and weighting schemes for the surveys from India and the United States. Our study in India used data from the Indian CTIS (nonprobability sample) and the CVoter survey (probability survey), while in the United States, we utilized the U.S. CTIS (nonprobability sample) and the Axios-Ipsos survey (probability survey) as data sources. Weighting for the CTIS surveys included design weights, individual-level nonresponse weights, and post-stratification weights (noncoverage weights). The CVoter survey and Axios-Ipsos survey, both considered probability surveys, used post-stratification methods to account for various sources of bias, including nonresponse bias. Post-stratification weights involved the use of external data to establish demographic benchmarks. The external data sources utilized in surveys include the following: the 2019 March CPS Supplement for the Axios-Ipsos survey (United States), the 2017 March CPS Supplement for the U.S. CTIS (United States), the most recent Census (2011) and national sample surveys for the CVoter survey (India), and the United Nations (UN) Population Division’s 2019 World Population Projection for the Indian CTIS (India).

Because we use multiple surveys from both India and the United States, each accompanied by recommended survey weights, we use Fig. 2 to summarize the various data sources and weights for the surveys we used in our study. For India, we used data from two types of surveys: a large nonprobability sample (CTIS) with an average weekly sample size of around 25,000, and a small probability survey conducted by the Center for Voting Options and Trends in Election Research (CVoter) with an average weekly sample size of about 2700. In contrast to a nonprobability sample, a probability survey is a well-designed survey where every individual in the population has a positive probability of selection and every sample of a given size has a known probability of selection (*3*, *23*). Probability surveys do not have to be equal probability samples but could include complex survey designs with multiple stages and oversampling of certain subgroups. Both nonprobability samples and probability surveys are subject to nonresponse biases (*24*). The weights used in both CTIS and the CVoter survey aimed to address the differences between the samples and the national adult population (Fig. 2). Facebook used a two-step method to mitigate both nonresponse and noncoverage biases in CTIS (*25*), while the CVoter survey only used post-stratification weights (*26*). Age and gender were incorporated into the post-stratification weight calculation for both surveys, while the CVoter survey additionally factored in education, income, social group, and rurality. For the United States, mirroring the research conducted by Bradley *et al.* (*13*), we leveraged data sourced from CTIS and the Axios-Ipsos survey. The Axios-Ipsos survey is a probability web survey using an address-based sampling method, with approximately 1000 respondents per wave (*27*). Much like the CVoter survey, it exclusively used post-stratification weights for weight calculations.

The rest of the paper is organized as follows. In Results, we first describe the surveyed populations. We then present analysis results for estimating overall vaccination rates in India, using CTIS and CVoter, similar to the study by Bradley *et al.* (*13*) for the United States. Finally, we consider three different estimands (i) to (iii) mentioned above and evaluate the extent of the Big Data Paradox for these estimands. We conclude with a discussion of our findings. In Materials and Methods, we describe the data sources we used for both India and the United States in more detail and present our analytical methods for the three estimands we consider. We first review the framework of error decomposition proposed by Meng (*9*) and adapt it to the differences in two means for estimands in (i) and (ii). For estimating vaccine hesitancy in (iii), we describe our model-based estimate that uses vaccine uptake as an auxiliary variable.

## RESULTS

### Characteristics of the survey respondents

#### Indian CTIS versus CVoter

The first half of Table 1 summarizes the unweighted and weighted demographics from both the CTIS and the CVoter survey in India, compared to the demographic data reported in the latest Indian Census (2011) reports. However, despite using age and gender in the weights, the CTIS weights could not recover the population distributions, resulting in the weighted sample still deviating from the general population. For example, the proportion of individuals aged between 25 and 44 years old in the weighted sample of the CTIS (53%) was closer to that in the general adult population (47%) when compared to the unweighted sample (63%). Similarly, the percentage of males in the weighted sample of the CTIS (66%) was more in line with the general adult population (52%) in contrast to the unweighted sample (84%). The residual discrepancy between the demographics reported in the Indian census and the weighted version of CTIS may stem from the disagreement between self-reported age and gender in the CTIS survey (used for calculating demographic distributions in our study) and the data obtained from the users’ Facebook profiles (used to compute the weights by Facebook) (*28*). In India, smartphones and social media accounts are often shared (*29*) and there is often intentional misreporting of gender on the public-facing Facebook profile (*30*). On the other hand, the weighted sample from CVoter closely aligns with the demographic distribution benchmarks in terms of age and gender. For instance, the proportion of males in the sample mirrors that of the Indian general adult population, accounting for 52%.

**Table 1.** Composition of the survey respondents and the sampling frames by demographic variables in India and the United States. The latest census (2011) and the March 2019 CPS Supplements were used for the census data in India and the United States, separately. The CTIS included respondents in India from 16 May 2021 to 18 September 2021 (*n* = 442,793), and respondents in the United States from 10 January 2021 to 15 May 2021 (*n* = 6,256,175). The CTIS used the adult Facebook Active User Base as its sampling frame, and we estimated the demographic distribution using Facebook advertising tools. However, demographic distributions for respondents in the Axios-Ipsos survey were not accessible.

Country	India						United States			
Data source	CTIS (25,000/week)			CVoter (2700/week)		Census	CTIS (250,000/week)			Census
Population	Unweighted respondents	Weighted respondents	Sampling frame	Unweighted respondents	Weighted respondents		Unweighted respondents	Weighted respondents	Sampling frame	
Gender*										
Female	16%	34%	24%	39%	48%	48%	67%	52%	56%	51%
Male	84%	66%	76%	61%	52%	52%	33%	46%	44%	49%
Age										
18–24 years	23%	21%	22%	19%	22%	22%	4%	11%	20%	12%
25–44 years	63%	53%	47%	54%	47%	47%	29%	33%	44%	34%
Above 45	14%	26%	31%	27%	31%	31%	67%	56%	36%	54%
Education										
Less than college	26%	26%	24%	86%	87%	87%	20%	21%	44%	39%
College and above	74%	74%	76%	14%	13%	13%	80%	79%	56%	61%
Location										
Rural	27%	25%		60%	70%	69%				
Urban	73%	75%		40%	30%	31%				
Proportion in total population			40%						87%	

*The survey participants provided self-reported gender information, whereas the census data reflects biological sex.

In terms of education and rurality, which were considered in the CVoter weights but not in the CTIS weights, the weighted proportions in the CVoter survey were also more in line with the census data. Specifically, the weighted proportions of individuals with at least a college education or residing in an urban area in the CVoter survey (13% and 30%, respectively) were closer to the corresponding values in the census data (13% and 31%, respectively), whereas the CTIS survey showed substantially higher proportions (74% and 75%, respectively). In general, the weighted CTIS sample is far from being representative of the Indian adult population, whereas the weighted CVoter sample closely mirrors the demographic distribution of the populace for the listed variables.

#### Indian CTIS versus U.S. CTIS

Table 1 also highlights a comparison of the demographic characteristics of the source population [adult Facebook Active User Base (FAUB)] in India and the United States with those of the weighted respondents from the CTIS. We calculated the demographic distributions of the source population using Facebook’s self-serve advertising tools, which provide the estimated population size for each distinct demographic subgroup (*31*). We used the March 2019 Current Population Survey (CPS) Supplement as the demographic benchmark for the United States. The proportion of adults that were Facebook active users in 2021 was smaller in India than in the United States (40% versus 87%). Among those who used Facebook actively, an overwhelming majority (76%) were males in India, whereas in the United States, the majority (56%) were females.

The weighted samples of the CTIS respondents in both India and the United States differed from the census demographic benchmarks with respect to the distribution of certain variables. However, the deviation was substantially more pronounced in India than in the United States. Specifically, the discrepancy in the proportion of female participants between the weighted respondents and the general adult population in India was larger than the corresponding difference in the United States. While the proportion of females in the weighted CTIS sample differed with the census by 1% in the United States, it differed by 14% in India. For demographic factors not used in the weights, for example, education, the difference in the proportion of participants with less than a college education between the weighted respondents and the general adult population in India was also notably larger compared to the United States (absolute difference 61% versus 18%). Because both gender and education are likely related to vaccination uptake (*32*–*34*), the estimate based on the weighted CTIS sample is likely to yield a biased estimate of vaccination rates in India.

### Characterizing the Big Data Paradox in India

#### Vaccine uptake in India

**Fig. 3.**
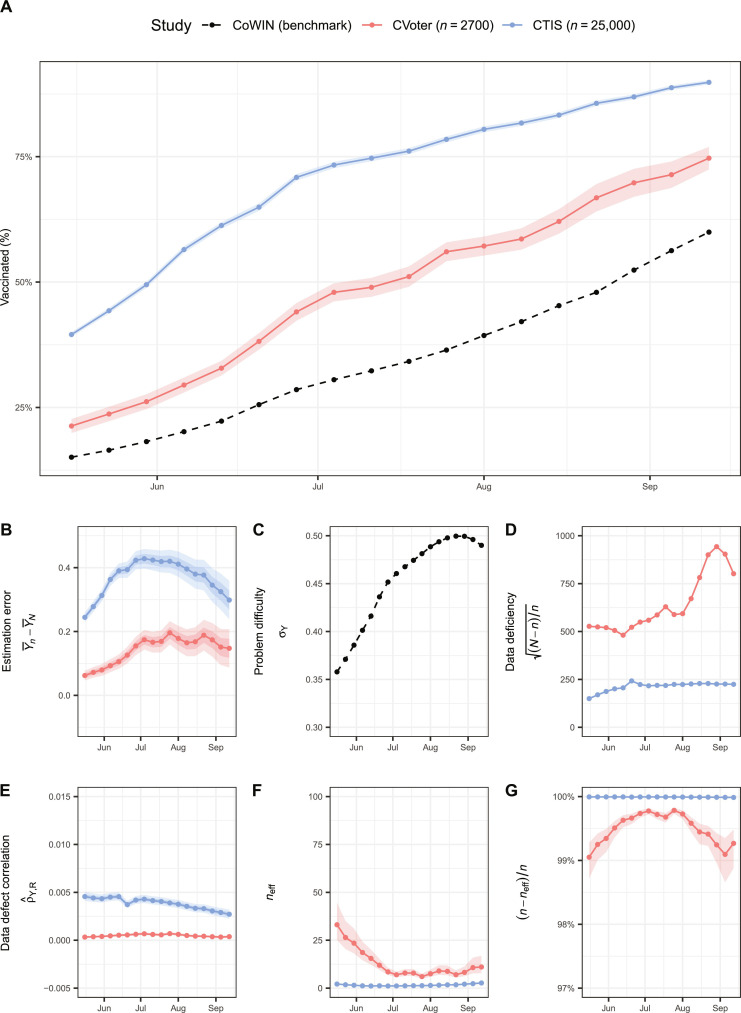
Decomposition of the estimation error and the effective sample size of vaccine uptake in India. (**A**) Weekly estimate, (**B**) estimation error, (**C**) inherent problem difficulty, (**D**) data deficiency, (**E**) data defect correlation, (**F**) effective sample size, and (**G**) reduction in the sample size of vaccine uptake among Indian adults from the CTIS (blue) and the CVoter survey (red) compared to the CoWIN benchmark (black) between 16 May and 18 September 2021. For (A), shaded bands show the classic 95% confidence intervals of the estimates. For (B) and (E), shaded bands show the ±5% and ±10% benchmark imprecision adjustments. For (F) and (G), shaded bands show the ±5% benchmark imprecision adjustments.

Both the weighted CTIS and CVoter reported a higher percentage of the adult population in India receiving their first dose of the COVID-19 vaccine from 16 May to 18 September 2021, compared to the benchmark (Fig. 3A). The benchmark in this study was sourced from the COVID Vaccine Intelligence Network (CoWIN) portal (*11*) The CTIS had a larger sample size than the CVoter (median 25,000 versus 2700); however, its estimate of the vaccination rate was more biased than that generated by the CVoter survey. The estimation errors from the CTIS exhibited a range from 25 to 45% with an average of 37%, whereas the CVoter survey showed a range from 5 to 20%, with an average of 14% (Fig. 3B). This finding is consistent with the Big Data Paradox, which suggests that a larger nonprobability sample (CTIS) may produce more biased estimates with narrower confidence intervals than a smaller designed survey (CVoter).

We conducted a decomposition of the estimation error in vaccine uptake into three components using Meng’s formula (*9*). These components include ddc (reflecting data quality), data deficiency (fraction of the population not included in the sample), and the inherent problem difficulty (variation in the vaccine uptake across the population) (*35*). The ddc captures the correlation between two random variables, the vaccination status of a participant, and a binary indicator denoting whether the data for this participant are recorded in the sample. Further details on this formula are reviewed in Materials and Methods. The values for inherent problem difficulty in both surveys were identical (Fig. 3C) because we assumed the national CoWIN data as the reference standard in both cases. However, the data deficiency values in the CVoter survey were comparatively higher than those in the CTIS (median, 588 versus 224; Fig. 3D), which can be attributed to differences in the original sample sizes (median, 2700 versus 25,000). The ddc magnitudes in the CVoter survey were lower than those in the CTIS (median, 0.0005 vs. 0.0040; Fig. 3E). This highlights a more pronounced selection bias in the self-selected nonprobability sample (CTIS) compared to the probability-designed survey (CVoter), consistent with our expectations.

The median weekly effective sample size of the CTIS was found to be 2 (Fig. 3F). This implies that in India, an SRS with a sample size of 2 could have the same MSE as observed from a survey with an original sample size of 25,000 (CTIS). The effective sample size in the CTIS was reduced by approximately 99.99% compared to the original sample size (Fig. 3G). The marked reduction in the sample size offers compelling evidence for the Big Data Paradox, which states that a nonprobability sample with a large sample size can be misleading when the population average is used as the estimand.

Additionally, the decrease in the effective sample size of the CVoter survey compared to its original sample size (median, 9 versus 2700) implies that selection bias due to missing responses may persist even in a probability survey. It is likely that the post-stratification weights used in CVoter could not fully correct for this nonresponse bias. The data recording mechanism in a probability survey can remain correlated with the outcome even after weighting, if the nonresponse weights are not correctly specified, leading to a nonzero ddc.

### Comparing the decomposition of estimation error in India and the United States

#### Comparison of nonprobability CTIS samples: India versus the United States

We evaluated the vaccine uptakes in the United States from 7 February to 15 May 2021, against those in India between 16 May and 18 September 2021, regarding the estimation error and its three components. These two periods are comparable in the sense that benchmark national vaccination rates observed at the start and conclusion of the periods chosen for the United States mirror those observed in India. Because of differences in access to vaccines and distribution policies, there was a temporal lag in vaccination uptake in the two countries. The choice was made to address that lag and make the two estimation problems more similar in their problem difficulty. The time frame of our investigation in the United States differs from that of the study by Bradley *et al.* (*13*), as described in Materials and Methods. The Indian and U.S. CTIS both overestimated the vaccine coverage among adults when compared to the benchmark data in each country (Fig. 1 and fig. S1). Nevertheless, the CTIS in India had larger estimation errors than the CTIS in the United States over time (median, 0.39 versus 0.16; Table 2). The larger differences between the estimated and benchmark values in India may be caused by the higher values of data deficiency in India compared to the United States (median, 224 versus median 34). Not only does India have a larger overall adult population, but the original sample sizes were also significantly smaller than those in the United States. The inherent problem difficulty levels in the Indian CTIS were comparable to those of the U.S. CTIS (median, 0.47 versus 0.49). This alignment results from our choice of the time periods we considered for each country, ensuring comparable national vaccination rates and similar SDs in both countries during the chosen periods. The values of ddc were smaller in the Indian version than in the U.S. version (median, 0.0040 versus 0.0079). This indicates that the selection bias in the U.S. CTIS was more substantial. Previously, we found that the sampling frame in the U.S. version covered a larger proportion of the entire population, and the respondents in the U.S. version were more representative of the general adult population considering certain variables. However, neither of these can ensure the correlation between the outcome of interest and the response indicator will be zero/negligible, which is in agreement with prior research (*13*).

**Table 2.** Estimation errors and their components of vaccine uptake from surveys in India and the United States. The benchmark data for India and the United States were from the CoWIN and the CDC, separately. Each continuous variable reported its mean, median, minimum, and maximum in the form of the mean, median [minimum, maximum].

Country	India (16 May to 18 September 18 2021)		United States (7 February to 15 May 2021)	
Survey	CTIS	CVoter survey	CTIS	Axios-Ipsos survey
Average sample size	25,000/week	2700/week	250,000/week	1000/wave
Benchmark (%) ${\bar{Y}}_{n}$	34.62, 33.24 [15.08, 59.95]		39.61, 40.07 [15.58, 61.91]	
Estimate (%) ${\bar{Y}}_{n}$	71.44, 75.40 [39.54, 89.82]	48.90, 50.02 [21.30, 74.70]	51.21, 52.92 [20.72, 76.91]	35.63, 30.50 [15.00, 64.00]
Estimation error (%) ${\bar{Y}}_{n}-{\bar{Y}}_{N}$	36.83, 38.50 [24.46, 42.80]	14.29, 15.99 [6.22, 19.60]	11.60, 12.85 [5.14, 15.62]	1.41, 0.98 [−0.95, 4.18]
Data deficiency $\sqrt{\frac{N-n}{n}}$	213, 224 [150, 242]	644, 588 [481, 943]	33, 33 [31, 39]	533, 535 [510, 554]
Problem difficulty σ*_Y_*	0.45, 0.47 [0.36, 0.50]		0.46, 0.49 [0.37, 0.50]	
Data defect correlation ${\hat{\rho }}_{Y,R}$	0.0038, 0.0040 [0.0027, 0.0046]	0.0005, 0.0005 [0.0003, 0.0007]	0.0073, 0.0079 [0.0045, 0.0091]	0.0001, 0.0000 [−0.0001, 0.0002]
Effective sample size *n_eff_*	1, 2 [1, 3]	13, 9 [6, 33]	20, 15 [10, 50]	677, 859 [138, 980]

#### Comparison of probability surveys: CVoter (India) versus Axios-Ipsos (the United States)

Regarding small probability surveys, it is noteworthy that the vaccination rate estimates obtained from the CVoter survey did not align as well with the population averages reported in the benchmark, in contrast to the Axios-Ipsos survey (Table 2). The quality of the probability survey can vary from country to country. Despite having similar values of data deficiency and inherent problem difficulty, the CVoter survey had much larger estimation errors compared to the Axios-Ipsos survey (median, 0.16 versus 0.01). This was possibly due to the CVoter survey having notably larger values of ddc (median, 0.0005 versus 0.0001). Although the CVoter survey used a probability-based sampling, the impact of nonresponse bias is a substantial concern even after weighting. It is plausible that the weights, which rely solely on post-stratification in terms of a few limited demographic variables, could not address the nonresponse bias completely (*24*). However, it is still better than the Indian CTIS in terms of estimation error, ddc, and effective sample size (Fig. 3). In addition, owing to a smaller sample size, CVoter does not overstate its accuracy and has a wider confidence interval compared to CTIS (Fig. 3).

### Evaluating the Big Data Paradox for other estimands beyond the mean

In the following sections, our aim is to assess the accuracy (regarding MSE) of the CTIS by shifting the estimand from the overall vaccination rate, known for the substantial estimation errors in both India and the United States, to others (Fig. 1).

#### Successive difference and relative successive difference in vaccine uptake

Change in vaccination rates (say daily or weekly) proved to be a valuable tool for monitoring the progress of the vaccination program during the pandemic (*36*). In this section, our chosen new estimands are the successive difference and relative successive difference in two population means over time. We evaluated the accuracy of the CTIS estimates for those two estimands by comparing them with the original estimand, using the metric of effective sample size, within the framework proposed by Meng (*9*). For the two new estimands, the effective sample size is also defined as the sample size of an SRS survey that has the same MSE as observed. Please refer to Materials and Methods for detailed derivations.

**Fig. 4.**
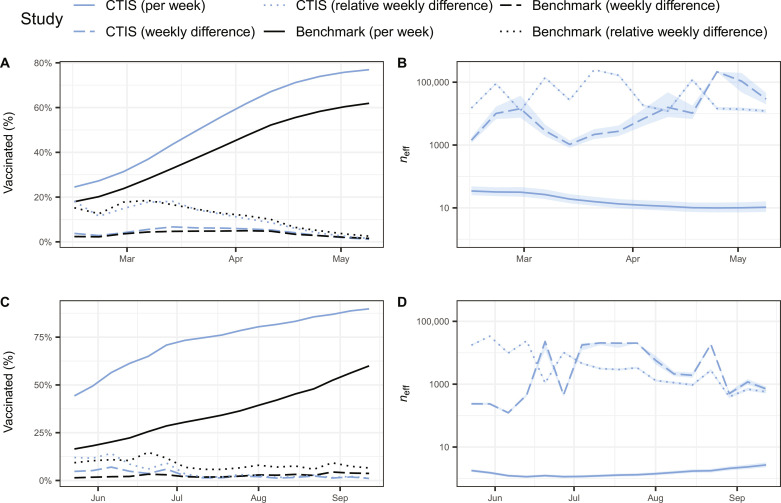
The successive difference and relative successive difference of vaccine uptake in the United States and India. (**A**) The estimate and (**B**) effective sample size of the overall rate (blue, solid), the successive difference (blue, dashed), and the relative successive difference (blue, dotted) of vaccine uptake among U.S. adults from the CTIS compared to the CDC benchmark (black, solid; black, dashed; and black, dotted) between 7 February and 15 May 2021. (**C**) The estimate and (**D**) effective sample size of the overall rate (blue, solid), the successive difference (blue, dashed), and the relative successive difference (blue, dotted) of vaccine uptake among Indian adults from the CTIS compared to the CoWIN benchmark (black, solid; black, dashed; and black, dotted) between 16 May and 18 September 2021. For (B) and (D), shaded bands show the ±5% benchmark imprecision adjustments.

During the time frame of 7 February to 15 May 2021, we checked the U.S. CTIS estimates of the vaccination rate difference and relative difference at consecutive weeks. Compared to the CDC benchmark, the median estimation errors of the difference and relative difference from the U.S. CTIS were 0.007 and −0.010, respectively. The median estimation error for the vaccination rate per week was 0.128 (Fig. 4A). The estimands based on the successive difference and relative successive difference yielded considerably larger effective sample sizes compared to the original estimand (median, 10,113 and 18,012 versus 15; Fig. 4B), representing a considerably higher proportion of the original sample size (median, 3.74% and 7.59% versus 0.01%).

In a similar manner, we examined the successive difference and relative successive difference in vaccination rates as estimated by the Indian CTIS from 16 May to 18 September 2021. In comparison with the CoWIN benchmark, the median estimation errors were −0.003 for the difference and −0.039 for the relative difference. The median estimation error for the weekly vaccination rate was 0.390 (Fig. 4C). Notably, the estimates of the successive difference and relative successive difference showcased substantially larger effective sample sizes when compared to the original estimate (median, 1922 and 2929 versus 2; Fig. 4D), accounting for a significantly higher proportion of the original sample size (median, 10.18% and 14.94% versus 0.01%). The findings in the United States and India indicate that the CTIS can provide more reliable estimates of the absolute and relative number of newly vaccinated people, compared to its estimate of cumulative vaccinated people.

#### Gender difference in vaccine uptake

Numerous studies from various countries have highlighted significant disparities in COVID-19 vaccine coverage across different demographic subgroups (*20*, *37*–*39*). Quick identification of these disparities can substantially contribute to achieving vaccination objectives and addressing health inequities. In this section, we focused on analyzing subgroup differences, using gender as an illustrative example. We introduced a formula for the error decomposition and effective sample size regarding the subgroup differences in means, drawing from the formulas regarding the overall mean proposed by Meng (*9*). The interpretations of the three components (ddc, data deficiency, and inherent problem difficulty) and the effective sample size remain the same as those in the original formulas. In this section, when calculating the overall vaccination rate in the CTIS, we excluded individuals who did not provide self-reported gender information. Detailed derivations are available in Materials and Methods.

Using the CTIS and the CDC benchmark data from 7 February to 15 May 2021, we investigated the difference in adult vaccine uptake average between females and males in the United States. While both the CTIS and the benchmark data indicated that females were more likely to be vaccinated during the study period, the CTIS underestimated the magnitude of the gender gap in vaccination rates compared to the benchmark (median difference, 0.05 versus 0.07; fig. S2).

**Fig. 5.**
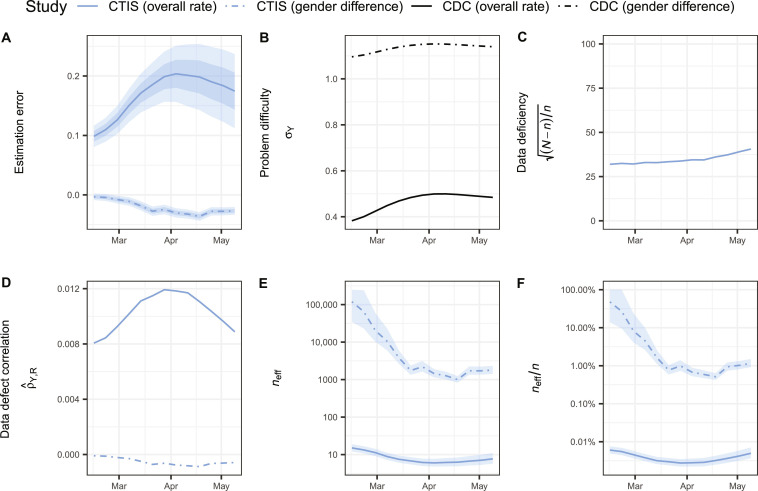
Decomposition of the estimation error and effective sample size of gender difference in vaccine uptake among U.S. adults. (**A**) Estimation error, (**B**) inherent problem difficulty, (**C**) data deficiency, (**D**) data defect correlation, (**E**) effective sample size, and (**F**) its proportion in the sample size of the overall rate (solid) and the gender difference (dot-dash) of vaccine uptake among U.S. adults from the CTIS (blue) compared to the CDC benchmark (black) between 7 February and 16 May 2021. For (A) and (D), shaded bands show the ±5% and ±10% benchmark imprecision adjustments. For (E) and (F), shaded bands show the ±5% benchmark imprecision adjustments.

Compared with the overall vaccination rates, the gender differences (females versus males) exhibited relatively smaller estimation errors (median, −0.03 versus 0.18; Fig. 5A). Of the three components of the estimation error, the inherent problem difficulty is not comparable across different estimands. The problem difficulty was naturally higher for the difference in two means compared to the overall vaccination rate due to the different scales of the outcomes (median SD over time, 1.14 versus 0.48; Fig. 5B). The values of data deficiency were identical in both scenarios as they are functions of the sample size and population size, which remain the same for a given survey (Fig. 5C). The ddc and effective sample size are standardized measures. The gender difference estimates had smaller magnitudes in terms of ddc (median, −0.006 versus 0.103), resulting in significantly greater effective sample sizes (median, 1999 versus 7) and less loss from the original sample size (median, 98.91% versus 99.99%), compared to the overall vaccination rate estimates (Fig. 5, D to F). The results suggest that a survey that is biased in estimating the overall rate can still provide a less biased estimate of the difference in means between subgroups.

#### Using vaccine uptake as auxiliary information for vaccine hesitancy

Estimation of vaccine uptake is an atypical situation where the true population mean ( ${\bar{Y}}_{n}$ ) is observed, thanks to national immunization records being available on the entire population. The availability of both sample and population means ensures that the estimation error can be calculated and the ddc can be estimated.

Analysis of vaccine hesitancy is a more typical situation where national data are not available; thus, the true population mean ( ${\bar{Y}}_{n}$ ) is unknown. We can, however, leverage the correlation between vaccine hesitancy and vaccine uptake, which has been highlighted in numerous studies (*40*, *41*), by incorporating vaccine uptake data as auxiliary information when estimating mean vaccine hesitancy.

We estimated vaccine hesitancy among U.S. adults between 10 January and 15 May 2021, using vaccine uptake data as auxiliary information. A beta regression model (*42*) was fit using data from the Axios-Ipsos survey with the outcome equal to the sample mean of vaccine hesitancy at each time *t* and a single covariate of the sample mean of vaccine uptake at that time. Predictions of vaccine hesitancy were then calculated using this model for both the Axios-Ipsos survey and the U.S. CTIS and used as inputs to a model-based estimate. The new estimator ( ${\bar{Y}}_{n,t}^{†}$ ) that leverages these predictions is given by

$${\bar{Y}}_{n,t}^{†}=\frac{1}{N_{t}}\sum_{i=1}^{N_{t}}\;\left[\frac{w_{i,t}R_{i,t}y_{i,t}}{w_{i,t}R_{i,t}}+\left(1-R_{i,t}\right){\hat{b}}_{t}\right]$$ (1)

At time *t*, *N_t_* represents the size of the finite population, *R*_*i*,*t*_ is a binary indicator (1 when the response is recorded) for individual *i*, *w*_*i*,*t*_ denotes the weight for individual *i*, and ${\hat{b}}_{t}$ is the estimated value derived from substituting the population average vaccine uptake to the aforementioned beta regression. Equation 1 is adapted from the model-assisted estimator (*43*), with the distinction that the design weight is not known in this scenario. For a detailed derivation, please refer to Materials and Methods.

Last, we performed the error decomposition (*9*) for both the naive estimate (using only the U.S. CTIS vaccine hesitancy data) and model-based estimates (using vaccine hesitancy data in the U.S. CTIS plus vaccine uptake data and the estimated relationship between the two) and compared their results in terms of the effective sample size.

**Fig. 6.**
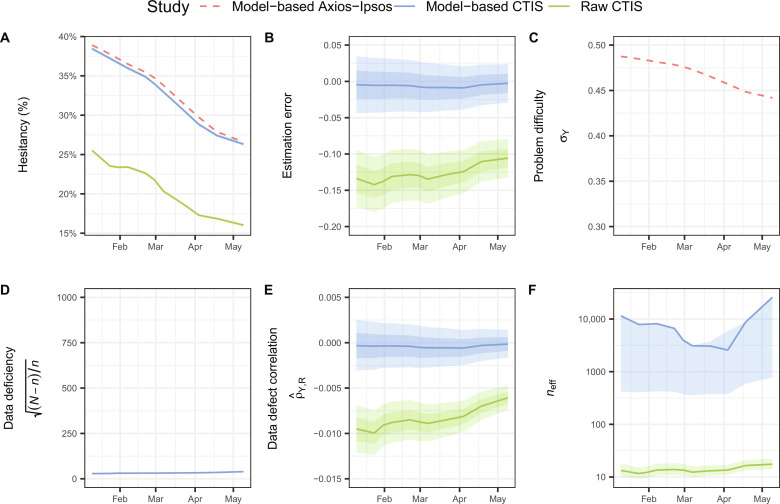
Decomposition of the estimation error and effective sample size of vaccine hesitancy in the United States. (**A**) The estimate, (**B**) estimation error, (**C**) inherent problem difficulty, (**D**) data deficiency, (**E**) data defect correlation, and (**F**) effective sample size of the vaccine hesitancy among U.S. adults from the model-based CTIS (blue) and original CTIS (green), compared to the model-based Axios-Ipsos benchmark (red) between 10 January and 15 May 2021. For (B) and (E), shaded bands show the ±5% and ±10% benchmark imprecision adjustments. For (F), shaded bands show the ±5% benchmark imprecision adjustments.

The Axios-Ipsos survey (used in the beta regression model) showed a strong correlation between vaccine hesitancy and vaccine uptake (pseudo *R*^2^ = 0.86). The CTIS survey underestimated the extent of vaccine unwillingness among U.S. adults when model-based estimates from the Axios-Ipsos survey were used as benchmarks (Fig. 6A). Vaccine hesitancy decreased over time in both surveys as the vaccine program progressed (Fig. 6A), which aligns with findings from previously published research (*44*). The estimates using vaccine uptake as auxiliary information exhibited smaller estimation errors than the original estimates (median, −0.01 versus −0.13; Fig. 6B). The effective sample sizes were larger in the model-based CTIS estimates than those in the original CTIS estimates (median, 7896 versus 13; Fig. 6F). When population-based data for an auxiliary variable, highly correlated with the variable of interest, are accessible, they aid in enhancing the accuracy of estimating the population average of the target variable.

## DISCUSSION

Our study serves as both a parallel and an extension to previous research on COVID-19 vaccination rates among adults in the United States using CTIS data (*13*). In Results, akin to the work by Bradley *et al.* (*13*), we first demonstrate that a large nonprobability sample (namely, CTIS) produces more biased estimates of vaccination uptake among Indian adults in comparison to a smaller probability survey (namely, CVoter). These findings emphasize that the Big Data Paradox holds for data from LMICs. In comparison to the U.S. CTIS results during a similar period of benchmark vaccination rates, the Indian CTIS exhibited higher values of estimation errors over time. The Indian version of theCTIS suffered more from data deficiency compared to the U.S. version, while it had smaller values of ddc. Additionally, our comparison of small probability surveys in India (namely, CVoter) and the United States (namely, Axios-Ipsos) shows that the former did not estimate the overall vaccination rates that closely matched the benchmark as the latter did. This observation underscores the vulnerability of probability surveys to nonresponse bias. Despite comparable response rates in the CVoter survey and the Axios-Ipsos survey (55% versus 50%), the nonresponse mechanism in the former remained only partially addressed and nonnegligible even after applying post-stratification weights. This emphasizes the importance of prioritizing data quality and creating reliable weights and robust estimates in survey design, consistent with the idea of the Big Data Paradox (*9*). We are not arguing that smaller is better, but rather that quality trumps quantity.

We then explored the important question of whether the CTIS data are useful for estimating alternative estimands as opposed to the overall vaccination rates. Using the same data sources as in the study by Bradley *et al.* (*13*), we observe that despite the obvious selection bias, the CTIS can provide more accurate estimates of (i) the successive difference and relative successive difference in vaccination rates, (ii) gender differences in vaccination rates, and (iii) vaccine hesitancy when using vaccination uptake as an auxiliary variable. For instance, the effective sample sizes for the successive difference (median, 10,113) were approximately 700 times greater than the effective sample sizes for the overall vaccination rate (median, 15). These results offer a more optimistic assessment of the CTIS data and similar large nonprobability samples, indicating that the Big Data Paradox is not inevitable for every estimand one may be interested in. Instead, we suggest that researchers study each estimand individually when analyzing self-selected samples. For instance, considering the lack of gender-specific vaccination data in India (*14*), the CTIS estimates can offer valuable insights into the differences in vaccination rates between females and males (fig. S3).

One may be interested in countries beyond India and the United States. To that end, using the CTIS global data, we expanded our examination of the estimation error in COVID-19 vaccination rates through the lens of the Big Data Paradox to span 85 additional countries. The results can be easily accessed through an interactive R Shiny application, which is available at the following link: https://3ogdqc-youqi-yang.shinyapps.io/LLPinVaccine/. More information about this tool is provided in section S6.

Kundu *et al.* (*45*) compared different weighting approaches to obtain bias-reduced inference when dealing with data that have substantial selection bias. Here, we did not pursue a more in-depth weighted analysis, as our objective was primarily to illustrate the prevalence of the Big Data Paradox across different populations and different estimands. It is important to acknowledge that creating correct weights for vaccine uptake is challenging due to the presence of numerous unmeasured individual variables that can potentially influence the response. The ddc ρ_*Y*,*R*_ gauges the relationship between the survey outcome (*Y*) and the response indicator (*R*). The response indicator denotes whether a response has been recorded, necessitating an individual to be first invited to the study and then respond to a given question at a given time. We can conceptualize the two processes as

$$\begin{array}{c} I\left(\text{Response}\;\text{is}\;\text{recorded}\;\text{for}\;a\;\text{participant}\right)=\\ I\left(\text{Participant}\;\text{included}\;\text{in}\;\text{the}\;\text{study}\right)\\ \times I\left(\text{Response}\;\text{is}\;\text{recorded}|\text{Participant}\;\text{is}\;\text{included}\right) \end{array}$$

In a probability survey, although we have knowledge of the first component through design probabilities (*23*), the nonresponse mechanism remains a major factor driving selection bias. Therefore, although CVoter achieves a seemingly representative sample based on the listed weighting variables—age, gender, education, and rurality—there could still be additional factors correlating with missingness and outcome. Conducting high-quality surveys, regardless of their scale, requires meticulous attention. Merely possessing a sampling frame and a probability-based survey design is not a universal remedy for mitigating bias in the resultant estimates.

There has been notable progress in finite population inference using nonprobability samples. Various methods have been introduced to integrate nonprobability and probability samples, including propensity score adjustment and calibration weighting methods (*46*, *47*). Additionally, a technique known as mass imputation has been utilized, involving the use of an independent nonprobability sample as a training dataset for imputation across all units within the probability sample (*48*). Data integration has also emerged as a promising avenue to link probability and nonprobability samples (*49*).

This study has several limitations. First, our comparison of estimator errors in probability and nonprobability samples was based solely on the framework of error decomposition proposed by Meng (*9*). Other methods have been suggested to quantify nonignorable selection mechanisms in nonprobability samples, including using indexes (*50*–*52*). Future research could investigate selection bias by utilizing these alternative methods and metrics. Second, as highlighted in Materials and Methods, potential measurement errors were present in both the CDC and CoWIN benchmarks when recording vaccination doses. Although we conducted a corresponding sensitivity analysis, a small deviation in the assumed benchmark for vaccination rate could result in a change in estimation error, along with the components (*53*). Third, in the framework of error decomposition from the study by Meng (*9*), we assumed that the response in the survey represented the true vaccination status of the respondent. However, it is possible that an unvaccinated individual might report being vaccinated due to social pressures, leading to differential misclassification. Therefore, it is crucial to account for uncertainty when utilizing this method, thus presenting a potential direction for future investigation. Dempsey (*54*) has proposed approaches for adjusting these measurement errors when applying error decomposition. Finally, the error decomposition proposed by Meng (*9*) is restrictive because it can only be applied to means. Further research can explore similar ideas in other parameters such as sample quantiles.

However, for countries like India, where limited individual-level data are available at the national level, one should do the best possible analysis of accessible data sources such as the CTIS. We hope our work will be helpful in enhancing the practitioners’ understanding of the Big Data Paradox and the alternatives that are available. We also hope our R Shiny app and accompanying code will encourage users to explore the global version of the CTIS, as most published analyses of the CTIS are United States–centric.

## MATERIALS AND METHODS

### Data source

#### Nonprobability samples used in our analysis

##### COVID-19 Trends and Impact Survey (India and the United States)

Facebook/Meta conducted the CTIS through their social media platform from April 2020 to June 2022 in partnership with the University of Maryland and Carnegie Mellon University (CMU) for global and U.S. versions, respectively (*15*, *28*). A stratified random sample of adults from the FAUB was invited daily to complete a cross-sectional questionnaire on COVID-related symptoms, exposures, outcomes, mental health, economic security, and demographics. The continuously adapted survey waves first launched queries about coronavirus vaccine uptake in January 2021. A potentially attractive feature of the CTIS is its large sample size. India and the U.S. survey data yielded approximately 25,000 and 250,000 respondents per week, respectively, in 2021. Facebook designed weights to adjust for the differences between the respondents and the general population in two components: individual-level nonresponse weights and post-stratification weights (*25*). For individual-level nonresponse weights, Facebook utilized an inverse propensity score weighting method to address the potential correlation between missingness and auxiliary information obtained from the user profile. This makes the survey respondent population more representative of the FAUB. For post-stratification weights, Facebook adjusted weights to match the respective national census benchmark in terms of age and gender. This was adjusted for those who are not active on Facebook or do not have internet access and made the survey more representative of the general population per country. Aggregated weighted responses are publicly available (*55*, *56*). The U.S. microdata were downloaded from CMU’s repository for daily tables with estimates and weights (*56*). In our study, we primarily used aggregated weighted data. However, for the new estimand (ii), we incorporated individual-level responses with weights provided by Facebook.

#### Probability surveys used in our analysis

##### CVoter COVID-19 tracker survey (India)

The CVoter administered the COVID-19 Tracker survey to measure symptoms and attitudes toward COVID-19 among Indian adults starting in March 2020 (*26*). The data available for analysis were last updated in July 2022. Team CVoter recruits a probability-based random sample of participants from the general public for a telephone interview per wave utilizing CATI. The survey introduced a vaccine uptake question in May 2021. The weekly average sample size was roughly 2700 in the time frame of our study. Analysis weights have been developed by Team CVoter to account for the differences between the respondents and the overall population in terms of age, gender, education, income, social group, and rurality. Detailed reports were made to be publicly available on their website (*57*) whereas our study used the individual-level data via collaboration with the CVoter team. Table 3 compares the study design characteristics of the two surveys we used for India.

**Table 3.** Comparisons of the survey designs in India. The benchmark data for vaccine uptake were sourced from the CoWIN.

	India	
	CTIS	CVoter survey
Survey type	Nonprobability	Probability
Target population	Indian adults	Indian adults
Sampling frame	Adult Facebook Active User Base	Adult telephone subscribers
Recruitment mode	Facebook newsfeed	Computer-assisted telephone interview
Interview mode	Online	Telephone
Average sample size per week	25,000	2700
Response rate	1%	55%
Vaccine uptake question	“Have you had a COVID-19 vaccination?”	“Have you got your COVID-19 vaccine shots?”
Vaccine uptake responses	“Yes”	“Yes, have got one shot/both shots of the vaccine”
Weighting variables	Age and gender	Age, gender, education, income, social group, and rurality
Sources for demographic benchmarks	UN Population Division 2019 World Population Projection	The latest census (2011) and national sample surveys (NSS) estimates

##### Axios-Ipsos coronavirus tracker (United States)

The analogously designed survey in the United States as used in the study by Bradley *et al.* (*13*) was the Axios-Ipsos Coronavirus tracker. Axios and Ipsos rolled out the bi-weekly survey to investigate COVID-19–like symptoms in the United States through the online Knowledge Panel starting in March 2020. The data available for analysis were last updated in December 2022 (*27*). The survey uses an address-based probabilistic sampling methodology, by using the delivery sequence file from the United States Poster Service. Ipsos provides a tablet and internet connection for the potential adult respondents who lack online connectivity, ensuring the sampling frame covers the entire adult population. The survey first inquired about vaccine uptake in December 2020. The number of respondents was roughly 1000 per wave (bi-weekly) throughout the study. Ipsos has developed weights to account for the differences between the respondents and the U.S. adult population in terms of age, gender, education, race/ethnicity, census region, metropolitan status, income, and partisanship. The weighted estimates were sourced from the topline portable document format (PDF) documents published on the Ipsos website (*27*). Table S1 compares design characteristics between the surveys we used for the United States. In our study, we quantified vaccine hesitancy as the proportion of respondents who indicated “Not very likely” or “Not at all likely” in response to the question assessing the likelihood of receiving the COVID-19 vaccine.

#### Benchmark official/government data

##### CoWIN (India)

We obtained the administrative vaccination counts from the CoWIN portal extracted via the website. CoWIN is an online portal for individual vaccine registration, playing a vital role in real-time nationwide vaccination monitoring (*11*). To align with the survey population, we must restrict the CoWIN vaccination counts to those given to adults. Given that India extended vaccine eligibility to citizens under 18 years old in 2022 (*58*), it is reasonable to utilize cumulative counts of the first-dose vaccine administration for the total population in 2021 as the gold standard counts. Potential recording errors could arise in the CoWIN database during the large-scale vaccination drive, such as merging dose certificates, unidentified records, and correction of vaccination dates. To account for potential imprecision in the benchmark data, we conducted a sensitivity analysis by multiplying the reported values from CoWIN by 0.9, 0.95, 1, 1.05, and 1.1.

##### CDC (United States)

The CDC benchmark data were extracted from reports disaggregated by age group and sex on the website (*10*). We used the total number of first-dose vaccines assigned to people aged 18 or older to match the survey population. We excluded those who did not report their age (<1%) in the analysis. In our comparison of vaccine coverage between genders, we additionally excluded individuals who did not report their gender information (<1%). The CDC has acknowledged a lag time between when an individual receives a vaccine and when it is reported to the CDC. We performed a similar adjustment for uncertainty as the CoWIN by scaling the CDC-reported numbers by a factor of 0.9, 0.95, 1, 1.05, and 1.1.

#### Demographic data (India and the United States)

For population-based demographic data used to calculate post-stratification weights, the Indian CTIS and the CVoter survey used the United Nations (UN) Population Division 2019 World Population Projection and the latest Indian Census (2011) with National Sample Surveys, respectively (*26*, *59*). The standard demographic distributions used by the U.S. CTIS and the Axios-Ipsos survey were from the March CPS Supplement, in 2017 and 2019, separately (*27*, *59*). Adult population sizes were extracted from the same sources in our study.

### Study period

Different illustrative examples in this paper have different time periods of analysis. Our analysis for the first objective, understanding the estimation error decomposition for national vaccination coverage in India, considers survey data from 16 May to 18 September 2021. We chose 16 May 2021 as our starting point because the CVoter survey launched the vaccination uptake question for the first time on that date. We chose 18 September 2021, as our ending point because the CoWIN benchmark estimated the proportion of the adult population that had received at least one dose of vaccine as 60% on that date. As a comparison, we studied the overall vaccination rate among U.S. adults between 7 February and 15 May 2021. We consider these two periods comparable as the benchmark vaccination rates we observed at the beginning and end of our analysis period in the United States were similar to those we observed in India (15% and 60%, respectively). For our second objective in studying new estimands (i) and (ii), we analyzed the successive differences, relative successive differences, and gender-specific subgroup differences in vaccination rates in each country over the same period as our first objective. Finally, we conducted an evaluation of COVID-19 vaccine hesitancy for scenario (iii) in the United States, focusing on the period from 10 January to 15 May 2021, the initial phase of the vaccination rollout where hesitancy estimates were most relevant.

### Error decomposition of the sample mean

Meng (*9*) provided a decomposition of estimation error in population mean and derived a formula for the resulting bias-adjusted effective sample size, *n_eff_*. For the sake of completeness, we review the main ideas here. Let *n* denote the size of the (potentially nonprobability) sample coming from a finite population of size *N*. Let ${\bar{Y}}_{n}$ be the sample average of the variable *Y*, and let ${\bar{Y}}_{N}$ be the population average. The discrepancy between ${\bar{Y}}_{n}$ and ${\bar{Y}}_{N}$ can be decomposed into three components using the following mathematical equation

$$\underset{\text{Estimation}\;\text{error}}{\underbrace{{\bar{Y}}_{n}-{\bar{Y}}_{N}}}=\underset{\text{Data}\;\text{defect}\;\text{correlation}}{\underbrace{\rho_{Y,R}}}\times \underset{\text{Data}\;\text{deficiency}}{\underbrace{\sqrt{\frac{N-n}{n}}}}\times \underset{\text{Inherent}\;\text{problem}\;\text{difficulty}}{\underbrace{\sigma_{Y}}}$$ (2)

The first component ρ_*Y*,*R*_ represents the population correlation between the variable *Y* and a binary recording indicator *R*, where we let *R* take the value 1 if we have recorded a value of *Y* in the sample and 0 otherwise. This measure is also called ddc because it directly addresses the bias due to the selection/recording mechanism by quantifying whether being included in the analytic sample depends on the outcome *Y*. The second component $\sqrt{\left(N-n\right)/n}$ represents data deficiency. The smaller the proportion of the entire population recorded, the larger the $\sqrt{\left(N-n\right)/n}$ , and the more challenging it becomes to estimate the ${\bar{Y}}_{N}$ accurately. The third component σ*_Y_*, the population SD of *Y*, represents the inherent problem difficulty by capturing random variation in *Y.*

The advantage of this representation as opposed to a standard bias-variance decomposition of the MSE (which is the squared version of the left-hand side in Eq. 2 by taking expectation with respect to the distribution of *R*) is that we can compare surveys with different recording mechanisms and sample sizes drawn from the same population for estimating the same target population quantity in terms of their accuracy.

In our investigation of the national coronavirus immunization rate, *n* is the survey sample size, *N* is the entire adult population size, and ${\bar{Y}}_{n}$ is the survey estimate of the vaccination rate. We have the rare and unique situation of knowing ${\bar{Y}}_{N}$ and σ*_Y_* from the national-level benchmark dataset. Thus, for different survey mechanisms (probability or nonprobability), the ddc ρ_*Y*,*R*_ can be estimated by plugging in the other values in Eq. 2. We denote the estimated value as ${\hat{\rho }}_{Y,R}$ , which is subject to potential measurement errors in both the surveys and the benchmark data. This quantity is not estimable from just sample data where we only observe *R* = 1.

The effective sample size (*n_eff_*) is defined as the size of an SRS drawn from the same population that will produce the same MSE as observed in the survey of interest with selection mechanism *R*. The formulas for the observed MSE (MSE*_R_*) and the MSE from the SRS (MSE*_SRS_*) are as follows

$${\text{MSE}}_{R}=E_{R}\left[\rho_{Y,R}^{2}\right]\times \frac{N-n}{n}\times \sigma_{Y}^{2}$$

$${\text{MSE}}_{\mathrm{SRS}}=\left[\frac{1}{N-1}\right]\times \frac{N-n_{\mathrm{eff}}}{n_{\mathrm{eff}}}\times \sigma_{Y}^{2}$$

By equating MSE*_R_* and MSE*_SRS_*, we have

$$n_{\mathrm{eff}}=\frac{\frac{n}{N-n}\times \frac{1}{E_{R}\left[\rho_{Y,R}^{2}\right]}}{\left(\frac{n}{N-n}\times \frac{1}{E_{R}\left[\rho_{Y,R}^{2}\right]}-1\right)\times \frac{1}{N}+1}$$

In our analysis of the vaccination rate, we approximate $E_{R}\left[\rho_{Y,R}^{2}\right]$ using the observed ${\hat{\rho }}_{Y,R}^{2}$ . Because *N* is large, we can assume that 1/*N* is close to 0, and simplify the formula for calculating the effective sample size as

$$n_{\mathrm{eff}}=\frac{n}{N-n}\times \frac{1}{{\hat{\rho }}_{Y,R}^{2}}$$ (3)

Equation 3 shows that having a large sample size *n* for the current survey does not necessarily guarantee accurate results if there is a substantial ddc ${\hat{\rho }}_{Y,R}$.

### Successive difference and relative successive difference in means

While the aforementioned section compares the survey mean ${\bar{Y}}_{n}$ and the benchmark mean ${\bar{Y}}_{N}$ , the method in this section compares survey estimates with the benchmark data when the targets are the difference and the relative difference in two means. Here, we consider differences in vaccination rates over successive time periods as our target. This formulation is similar to the one in the study by Dempsey (*54*) for measuring the change in COVID-19 infection rates. Let ${\bar{Y}}_{n,t}$ be the survey average of the variable *Y* at time *t*, and let ${\bar{Y}}_{N,t}$ be the population average at the same time *t*. In this scenario, the difference in the survey average of the variable *Y* from time *t* − 1 to *t* is represented by ${\bar{Y}}_{n,t}-{\bar{Y}}_{n,t-1}$ . The corresponding relative difference is represented by $\left({\bar{Y}}_{n,t}-{\bar{Y}}_{n,t-1}\right)/{\bar{Y}}_{n,t-1}$ . The difference in the population average from time *t* − 1 to *t* is represented by ${\bar{Y}}_{N,t}-{\bar{Y}}_{N,t-1}$ , and the corresponding relative difference is represented by $\left({\bar{Y}}_{N,t}-{\bar{Y}}_{N,t-1}\right)/{\bar{Y}}_{N,t-1}$ . Let $\sigma_{Y_{t}}^{2}$ be the population variance of *Y* at time *t*. Define the effective sample size *n_eff_* as the size of two SRS samples that can generate the observed MSE for the successive difference and relative successive difference. To simplify the analysis, we assume that both SRS samples have an equal sample size. We derive the following formulas for calculating the effective sample sizes in regard to the difference and relative difference at consecutive times. See sections S1 and S2 for a detailed calculation.

$$\text{Difference}\;\left(n_{\mathrm{eff}}\right)=\frac{\sigma_{Y_{t-1}}^{2}+\sigma_{Y_{t}}^{2}}{{\left[\left({\bar{Y}}_{n,t}-{\bar{Y}}_{n,t-1}\right)-\left({\bar{Y}}_{N,t}-{\bar{Y}}_{N,t-1}\right)\right]}^{2}}$$ (4)

$$\text{Relative}\;\text{difference}\;\left(n_{\mathrm{eff}}\right)=\frac{{\left(\frac{{\bar{Y}}_{N,t}}{{\bar{Y}}_{N,t-1}}\right)}^{2}\left[\frac{\sigma_{Y_{t-1}}^{2}}{{\left({\bar{Y}}_{N,t-1}\right)}^{2}}+\frac{\sigma_{Y_{t}}^{2}}{{\left({\bar{Y}}_{N,t}\right)}^{2}}\right]}{{\left[\frac{{\bar{Y}}_{n,t}-{\bar{Y}}_{n,t-1}}{{\bar{Y}}_{n,t-1}}-\frac{{\bar{Y}}_{N,t}-{\bar{Y}}_{N,t-1}}{{\bar{Y}}_{N,t-1}}\right]}^{2}}$$ (5)

To represent the above two equations in terms of the three components of the estimation error, we can use Eq. 2 to decompose the estimation error at different time points, namely, time *t* − 1 and *t*. Denote *n_t_* as the sample size of the survey conducted during time *t*, and denote *N_t_* as the population size during time *t*. Additionally, denote ρ_*Y_t_*,*R_t_*_ as the ddc at time *t*. Expanding the equations yields the following expressions. See section S3 and section S4 for a detailed calculation.

$$\begin{array}{c} \text{Difference}\left(n_{\mathrm{eff}}\right)=\\ \frac{\sigma_{Y_{t-1}}^{2}+\sigma_{Y_{t}}^{2}}{{\left[{\hat{\rho }}_{Y_{t},R_{t}}\times \sqrt{\frac{N_{t}\;-\;n_{t}}{n_{t}}}\times \sigma_{Y_{t}}-{\hat{\rho }}_{Y_{t-1},R_{t-1}}\times \sqrt{\frac{N_{t-1}-n_{t-1}}{n_{t-1}}}\times \sigma_{Y_{t-1}}\right]}^{2}} \end{array}$$

$$\begin{array}{c} \text{Relative}\;\text{difference}\;\left(n_{\mathrm{eff}}\right)=\\ \frac{\left[\frac{\sigma_{Y_{t-1}}^{2}}{{\left({\bar{Y}}_{N,t-1}\right)}^{2}}+\frac{\sigma_{Y_{t}}^{2}}{{\left({\bar{Y}}_{N,t}\right)}^{2}}\right]}{{\left[{\hat{\rho }}_{Y_{t},R_{t}}\times \sqrt{\frac{N_{t}-n_{t}}{n_{t}}}\times \frac{\sigma_{Y_{t}}}{{\bar{Y}}_{N,t}}-{\hat{\rho }}_{Y_{t-1},R_{t-1}}\times \sqrt{\frac{N_{t-1}-n_{t-1}}{n_{t-1}}}\times \frac{\sigma_{Y_{t-1}}}{{\bar{Y}}_{N,t-1}}\right]}^{2}}\times \\ \frac{1}{{\left[1-{\hat{\rho }}_{Y_{t-1},R_{t-1}}\times \sqrt{\frac{N_{t-1}-n_{t-1}}{n_{t-1}}}\times \frac{\sigma_{Y_{t-1}}}{{\bar{Y}}_{N,t-1}}\right]}^{2}} \end{array}$$

### Subgroup difference in means

In this section, we present a method for evaluating the accuracy of estimating mean differences between two subgroups following the decomposition approach in the study by Meng (*9*). Let ${\bar{Y}}_{n_{g}}$ be the survey average of the variable *Y* in the subgroup *g*, and let ${\bar{Y}}_{N_{g}}$ be the population average in the same subgroup. Let *n_g_* be the sample size of the survey conducted in the subgroup *g*, and let *N_g_* be the population size of the subgroup *g*. For simplicity, we assume that the general population can be explicitly divided into two partitions: group I and group II, *g* = I, II. To differentiate between these groups, we have created a binary indicator, represented by the variable *G*. If a response belongs to group II, the value of *G* will be 1. If a response belongs to group I, the value of *G* will be 0. Define a new variable *Y*^∗^, which is a combination of the variable *Y* and the indicator *G*, *Y*^∗^ = *Y* × *G* − *Y* × (1 − *G*). The revised formula for the decomposition of the estimation error can be written as follows

$$\left({\bar{Y}}_{n_{\text{II}}}-{\bar{Y}}_{n_{I}}\right)-\left({\bar{Y}}_{N_{\text{II}}}-{\bar{Y}}_{N_{I}}\right)=\rho_{Y^{*},R^{*}}\times \sqrt{\frac{\left(N_{I}+N_{\text{II}}\right)-\left(n_{I}+n_{\text{II}}\right)}{n_{I}+n_{\text{II}}}}\times \sigma_{Y^{*}}$$ (6)

where *R*^∗^ is a binary variable indicating if *Y*^∗^ is recorded. The focus of our study is to investigate the difference in vaccination rates between genders. In this context, we denote the estimated vaccination rate among females from the CTIS as ${\bar{Y}}_{n_{\text{II}}}$ , and the estimated vaccination rate among males from the CTIS as ${\bar{Y}}_{n_{I}}$ . The calculation of σ_*Y*^∗^_ is presented in section S5. We can then estimate ρ_*Y*^∗^,*R*^∗^_ by substituting the known values from benchmark data into Eq. 6 and solving it. The interpretation of each factor is the same as that in the original formula.

The revised formula for calculating the bias-adjusted effective sample size can be written as follows

$$n_{\mathrm{eff}}=\frac{n_{I}+n_{\text{II}}}{\left(N_{I}+N_{\text{II}}\right)-\left(n_{I}+n_{\text{II}}\right)}\times \frac{1}{{\left({\hat{\rho }}_{Y^{*},R^{*}}\right)}^{2}}$$ (7)

### Use as auxiliary information

In this section, we use the decomposition framework for assessing the estimation accuracy of variable *Y* with the assistance of auxiliary variable *X* correlated with *Y*. Deville and Sarndal (*43*) proposed the linear generalized regression estimator, which involves modeling the relationship between the variable of interest and the auxiliary variable. This method was subsequently extended to situations involving nonlinear relationships between variables (*60*).

In this framework, we introduce a response indicator, which is represented by the variable *R*. When this indicator takes on a value of 1, it signifies a recorded response. We use π to denote the response probability, specifically defined as π = Pr (*R* = 1) = *E*(π). Additionally, *N_t_* corresponds to the population size at time *t*. The formula for calculating the updated estimate ${\bar{Y}}_{n,t}^{†}$ using auxiliary information is then expressed as

$${\bar{Y}}_{n,t}^{†}=\frac{1}{N_{t}}\sum_{i=1}^{N_{t}}\;\left[\frac{R_{i,t}y_{i,t}}{\pi_{i,t}}+\left(1-\frac{R_{i,t}}{\pi_{i,t}}\right){\hat{y}}_{i,t}\right]$$ (8)

For outcomes not recorded within the sample, imputed values denoted ${\hat{y}}_{i,t}$ are used. This imputation depends on the relationship between the auxiliary variable (*X*) and the variable of interest (*Y*). It is worth emphasizing that the validity of Eq. 8 relies on knowledge of the individual response probability (π) or correct specification of the imputation model, i.e., a doubly robust estimator (*61*).

However, in our specific analysis, the probability (π) is not readily available. Consequently, the updated estimate ${\bar{Y}}_{n,t}^{†}$ within this study is characterized as “model-based” rather than “model-assisted.” This distinction arises from our estimation of the response probability through the use of weights as shown in Eq. 1. It is reasonable to anticipate a more accurate estimate of this probability when derived from a probability survey compared to a nonprobability sample.

Both the U.S. CTIS and Axios-Ipsos survey inquired about vaccine uptake and vaccine hesitancy simultaneously during the study period. Given that the sample average of vaccine hesitancy is between 0 and 1, beta regression is used to estimate the relationship (*42*). Here, we fit the data from the Axios-Ipsos survey, which is a probability survey. Denote the average of vaccine uptake at time *t* as *a_t_*, and denote the average of vaccine hesitancy at time *t* as *b_t_*. The model assumes that the outcome follows a beta distribution with a mean of μ*_t_* and a precision of ϕ. The equation is as follows

$$\text{log}\left(\frac{\mu_{t}}{1-\mu_{t}}\right)=\beta_{0}+\beta_{1}a_{t},b_{t}∼\text{Beta}\left(\mu_{t},\phi \right)$$ (9)

Parameter estimates are used to compute imputation values for Eq. 8, i.e., ${\hat{y}}_{i,t}$ , by substituting the population average vaccine uptake (*a_t_*) from the benchmark data at time *t*. This yields a single imputation value for all individuals at time *t*, which is denoted as ${\hat{b}}_{t}$ in Eq. 1. The analysis is performed for both the Axios-Ipsos survey and the U.S. CTIS separately.

To apply the error decomposition framework in means, as proposed by Meng (*9*), we make the assumption that the model-based estimate ${\bar{Y}}_{n,t}^{†}$ obtained from the Axios-Ipsos survey serves as the benchmark for measuring vaccine hesitancy among U.S. adults. We can then compare the original estimate ${\bar{Y}}_{n,t}$ with the model-based estimate ${\bar{Y}}_{n,t}^{†}$ from the CTIS to decompose the components of the estimation error and the effective sample size. Similarly, we performed an adjustment for uncertainty by scaling the Axios-Ipsos survey’s estimates by a factor of 0.9, 0.95, 1, 1.05, and 1.1.

## Supplementary Materials

### This PDF file includes:

Sections S1 to S6 Figs. S1 to S3 Table S1

## References

[1] R. Sammut, O. Griscti, I. J. Norman, Strategies to improve response rates to web surveys: A literature review. Int. J. Nurs. Stud. 123, 104058 (2021). 34454334 10.1016/j.ijnurstu.2021.104058

[2] N. Keiding, T. A. Louis, Perils and potentials of self-selected entry to epidemiological studies and surveys. J. R. Stat. Soc. Ser. A Stat. Soc. 179, 319–376 (2016).

[3] R. J. Little, Survey sampling: Past controversies, current orthodoxy, and future paradigms, in *Past, Present, and Future of Statistical Science* (CRC Press, Taylor & Francis Group, 2014), pp. 413–428.

[4] J. Bethlehem, Selection bias in web surveys. Int. Stat. Rev. 78, 161–188 (2010).

[5] M. Schonlau, A. van Soest, A. Kapteyn, M. Couper, Selection bias in web surveys and the use of propensity scores. Sociol. Methods Res. 37, 291–318 (2009).

[6] R. M. Groves, L. Lyberg, Total survey error: Past, present, and future. Public Opin. Q. 74, 849–879 (2010).

[7] J. Daikeler, M. Bošnjak, K. L. Manfreda, Web versus other survey modes: An updated and extended meta-analysis comparing response rates. J. Surv. Stat. Methodol. 8, 513–539 (2020).

[8] L. H. Nguyen, A. D. Joshi, D. A. Drew, J. Merino, W. Ma, C.-H. Lo, S. Kwon, K. Wang, M. S. Graham, L. Polidori, C. Menni, C. H. Sudre, A. Anyane-Yeboa, C. M. Astley, E. T. Warner, C. Y. Hu, S. Selvachandran, R. Davies, D. Nash, P. W. Franks, J. Wolf, S. Ourselin, C. J. Steves, T. D. Spector, A. T. Chan, COPE Consortium, Self-reported COVID-19 vaccine hesitancy and uptake among participants from different racial and ethnic groups in the united states and united kingdom. Nat. Commun. 13, 636 (2022). 35105869 10.1038/s41467-022-28200-3 PMC8807721

[9] X.-L. Meng, Statistical paradises and paradoxes in big data (i): Law of large populations, big data paradox, and the 2016 US presidential election. Ann. Appl. Stat. 12, 685–726 (2018).

[10] CDC, Data definitions for COVID-19 vaccinations in the United States (2023); https://www.cdc.gov/coronavirus/2019-ncov/vaccines/reporting-vaccinations.html.

[11] Government of India, CoWIN dashboard (2020); https://dashboard.cowin.gov.in/.

[12] E. Dong, H. Du, L. Gardner, An interactive web-based dashboard to track COVID-19 in real time. Lancet Infect. Dis. 20, 533–534 (2020). 32087114 10.1016/S1473-3099(20)30120-1 PMC7159018

[13] V. C. Bradley, S. Kuriwaki, M. Isakov, D. Sejdinovic, X.-L. Meng, S. Flaxman, Unrepresentative big surveys significantly overestimated US vaccine uptake. Nature 600, 695–700 (2021). 34880504 10.1038/s41586-021-04198-4 PMC8653636

[14] R. Grieve, Y. Yang, S. Abbott, G. R. Babu, M. Bhattacharyya, N. Dean, S. Evans, N. Jewell, S. M. Langan, W. Lee, G. Molenberghs, L. Smeeth, E. Williamson, B. Mukherjee, The importance of investing in data, models, experiments, team science, and public trust to help policymakers prepare for the next pandemic. PLOS Glob. Public Health 3, e0002601 (2023). 38032861 10.1371/journal.pgph.0002601 PMC10688710

[15] J. A. Salomon, A. Reinhart, A. Bilinski, E. J. Chua, W. La Motte-Kerr, M. M. Rönn, M. B. Reitsma, K. A. Morris, S. LaRocca, T. H. Farag, F. Kreuter, R. Rosenfeld, R. J. Tibshirani, The US COVID-19 trends and impact survey: Continuous real-time measurement of COVID-19 symptoms, risks, protective behaviors, testing, and vaccination. Proc. Natl. Acad. Sci. U.S.A. 118, e2111454118 (2021). 34903656 10.1073/pnas.2111454118 PMC8713763

[16] J. M. Kush, E. Badillo-Goicoechea, R. J. Musci, E. A. Stuart, Teachers’ mental health during the covid-19 pandemic. Educ. Res. 51, 593–597 (2022). 38603417 10.3102/0013189X221134281 PMC9666408

[17] C. Lupton-Smith, E. Badillo-Goicochea, T.-H. Chang, H. Maniates, K. E. Riehm, I. Schmid, E. A. Stuart, Factors associated with county-level mental health during the COVID-19 pandemic. J. Community Psychol. 50, 2431–2442 (2022). 34969152 10.1002/jcop.22785 PMC9015572

[18] S. D. McCabe, E. A. Hammershaimb, D. Cheng, A. Shi, D. Shyr, S. Shen, L. D. Cole, J. R. Cataldi, W. Allen, R. Probasco, B. Silbermann, F. Zhang, R. Marsh, M. A. Travassos, X. Lin, Unraveling attributes of COVID-19 vaccine hesitancy and uptake in the U.S.: A large nationwide study. medRxiv 2125498 [Preprint] (2021). 10.1101/2021.04.05.21254918. PMC10209066 37225748

[19] Q. C. Nguyen, I. Yardi, F. X. M. Gutierrez, H. Mane, X. Yue, Leveraging 13 million responses to the U.S. COVID-19 trends and impact survey to examine vaccine hesitancy, vaccination, and mask wearing, january 2021-february 2022. BMC Public Health 22, 1911 (2022). 36229804 10.1186/s12889-022-14286-3 PMC9559553

[20] A. D. McNaghten, N. T. Brewer, M.-C. Hung, P.-J. Lu, D. Daskalakis, N. Abad, J. Kriss, C. Black, E. Wilhelm, J. T. Lee, A. Gundlapalli, J. Cleveland, L. Elam-Evans, K. Bonner, J. Singleton, COVID-19 vaccination coverage and vaccine confidence by sexual orientation and gender identity—United States, August 29–October 30, 2021. MMWR Morb. Mortal. Wkly Rep. 71, 171–176 (2022). 35113846 10.15585/mmwr.mm7105a3 PMC8812836

[21] C. Wang, B. Han, T. Zhao, H. Liu, B. Liu, L. Chen, M. Xie, J. Liu, H. Zheng, S. Zhang, Y. Wang, N. Huang, J. Du, Y.-Q. Liu, Q.-B. Lu, F. Cui, Vaccination willingness, vaccine hesitancy, and estimated coverage at the first round of COVID-19 vaccination in China: A national cross-sectional study. Vaccine 39, 2833–2842 (2021). 33896661 10.1016/j.vaccine.2021.04.020 PMC8043613

[22] U. Kohler, F. Kreuter, E. A. Stuart, Nonprobability sampling and causal analysis. Annu. Rev. Stat. Appl. 6, 149–172 (2019).

[23] R. V. Craiu, R. Gong, X.-L. Meng, Six statistical senses. Annu. Rev. Stat. Appl. 10, 699–725 (2023).

[24] M. A. Bailey, A new paradigm for polling. Harvard Data Sci. Rev. 5, (2023).

[25] N. Barkay, C. Cobb, R. Eilat, T. Galili, D. Haimovich, S. LaRocca, K. Morris, T. Sarig, Weights and methodology brief for the COVID-19 symptom survey by University of Maryland and Carnegie Mellon University, in partnership with Facebook. arXiv:2009.14675 [cs.SI] (2020).

[26] Team CVoter, COVID-19 tracker surveys in India (2020); https://cvoterindia.com/wp-content/uploads/2020/Covid_Tracker_Methodology_Note.pdf.

[27] C. Jackson, M. Newall, J. Yi, Axios ipsos coronavirus index (2020); https://www.ipsos.com/en-us/news-polls/axios-ipsos-coronavirus-index.

[28] C. M. Astley, G. Tuli, K. A. Mc Cord, E. L. Cohn, B. Rader, T. J. Varrelman, S. L. Chiu, X. Deng, K. Stewart, T. H. Farag, K. M. Barkume, S. LaRocca, K. A. Morris, F. Kreuter, J. S. Brownstein, Global monitoring of the impact of the COVID-19 pandemic through online surveys sampled from the facebook user base. Proc. Natl. Acad. Sci. U.S.A. 118, e2111455118 (2021). 34903657 10.1073/pnas.2111455118 PMC8713788

[29] ASER Centre, Annual status of education report (2021) [accessed 9 November 2023]; https://asercentre.org/.

[30] Delphi Research Group, COVID-19 trends and impact survey: Survey limitations (2020) [accessed 9 November 2023]; https://cmu-delphi.github.io/delphi-epidata/symptom-survey/limitations.html.

[31] Facebook, Facebook self-serve advertising tools (2021); https://www.facebook.com/business/goals/run-facebook-ad-campaigns.

[32] V. J. Hall, S. Foulkes, A. Saei, N. Andrews, B. Oguti, A. Charlett, E. Wellington, J. Stowe, N. Gillson, A. Atti, J. Islam, I. Karagiannis, K. Munro, J. Khawam, M. A. Chand, C. S. Brown, M. Ramsay, J. Lopez-Bernal, S. Hopkins, COVID-19 vaccine coverage in health-care workers in england and effectiveness of BNT162b2 mRNA vaccine against infection (SIREN): A prospective, multicentre, cohort study. Lancet 397, 1725–1735 (2021). 33901423 10.1016/S0140-6736(21)00790-X PMC8064668

[33] T. Nassiri-Ansari, P. Atuhebwe, A. S. Ayisi, S. Goulding, M. Johri, P. Allotey, N. Schwalbe, Shifting gender barriers in immunisation in the COVID-19 pandemic response and beyond. Lancet 400, 24 (2022). 10.1016/S0140-6736(22)01189-8 PMC9246460 35780789

[34] K. Viswanath, M. Bekalu, D. Dhawan, R. Pinnamaneni, J. Lang, R. McLoud, Individual and social determinants of COVID-19 vaccine uptake. BMC Public Health 21, 818 (2021). 33910558 10.1186/s12889-021-10862-1 PMC8081000

[35] F. Kreuter, What surveys really say. Nature 600, 614–615 (2021). 34880485 10.1038/d41586-021-03604-1

[36] J. Diesel, N. Sterrett, S. Dasgupta, J. L. Kriss, V. Barry, K. Vanden Esschert, A. Whiteman, B. L. Cadwell, D. Weller, J. R. Qualters, L. Harris, A. Bhatt, C. Williams, L. M. Fox, D. Meaney Delman, C. L. Black, K. E. Barbour, COVID-19 vaccination coverage among adults—United States, December 14, 2020–May 22, 2021. MMWR Morb. Mortal. Wkly Rep. 70, 922–927 (2021). 34166331 10.15585/mmwr.mm7025e1 PMC8224863

[37] R. Saelee, E. Zell, B. P. Murthy, P. Castro-Roman, H. Fast, L. Meng, L. Shaw, L. Gibbs-Scharf, T. Chorba, L. Q. Harris, N. Murthy, Disparities in COVID-19 vaccination coverage between urban and rural counties—United States, December 14, 2020–January 31, 2022. Morb. Mortal. Wkly. Rep. 71, 335–340 (2022). 10.15585/mmwr.mm7109a2 PMC8893338 35239636

[38] K. Muhsen, W. Na’aminh, Y. Lapidot, S. Goren, Y. Amir, S. Perlman, M. S. Green, G. Chodick, D. Cohen, A nationwide analysis of population group differences in the COVID-19 epidemic in Israel, February 2020–February 2021. Lancet Reg. Health Eur. 7, 100130 (2021). 34109321 10.1016/j.lanepe.2021.100130 PMC8177966

[39] M. Perry, A. Akbari, S. Cottrell, M. B. Gravenor, R. Roberts, R. A. Lyons, S. Bedston, F. Torabi, L. Griffiths, Inequalities in coverage of COVID-19 vaccination: A population register based cross-sectional study in wales, uk. Vaccine 39, 6256–6261 (2021). 34544601 10.1016/j.vaccine.2021.09.019 PMC8423991

[40] M. Chutiyami, D. Salihu, U. M. Bello, S. J. Winser, A. A. Gambo, H. Sabo, A. M. Kolo, H. A. Jalo, A. S. Muhammad, F. A. Mahmud, K. K. Adeleye, O. M. Azubuike, I. M. Bukola, P. Kannan, Are fear of COVID-19 and vaccine hesitancy associated with COVID-19 vaccine uptake? A population-based online survey in Nigeria. Vaccines 10, 1271 (2022). 36016160 10.3390/vaccines10081271 PMC9415607

[41] M. S. Rane, S. Kochhar, E. Poehlein, W. You, M. M. Robertson, R. Zimba, D. A. Westmoreland, M. L. Romo, S. G. Kulkarni, M. Chang, A. Berry, A. M. Parcesepe, A. R. Maroko, C. Grov, D. Nash, Determinants and trends of COVID-19 vaccine hesitancy and vaccine uptake in a national cohort of US adults: A longitudinal study. Am. J. Epidemiol. 191, 570–583 (2022). 34999751 10.1093/aje/kwab293 PMC8755394

[42] S. L. Ferrari, F. Cribari-Neto, Beta regression for modelling rates and proportions. J. Appl. Stat. 31, 799–815 (2004).

[43] J.-C. Deville, C.-E. Sarndal, Calibration estimators in survey sampling. J. Am. Stat. Assoc. 87, 376–382 (1992).

[44] M. Daly, A. Jones, E. Robinson, Public trust and willingness to vaccinate against COVID-19 in the US from October 14, 2020, to March 29, 2021. JAMA 325, 2397–2399 (2021). 34028495 10.1001/jama.2021.8246 PMC8145162

[45] R. Kundu, X. Shi, J. Morrison, B. Mukherjee, A framework for understanding selection bias in real-world healthcare data. *J. Roy. Stat. Soc. Ser. A: Stat. Soc*. (2024). 10.1093/jrsssa/qnae039. PMC11393555 39281782

[46] M. R. Elliott, R. Valliant, Inference for nonprobability samples. Stat. Sci. 32, 249–264 (2017).

[47] Y. Chen, P. Li, C. Wu, Doubly robust inference with nonprobability survey samples. J. Am. Stat. Assoc. 115, 2011–2021 (2020).

[48] J. K. Kim, S. Park, Y. Chen, C. Wu, Combining non-probability and probability survey samples through mass imputation. J. R. Stat. Soc. Ser. A Stat. Soc. 184, 941–963 (2021).

[49] S. Yang, J. K. Kim, Statistical data integration in survey sampling: A review. Jpn. J. Stat. Data Sci. 3, 625–650 (2020).

[50] R. R. Andridge, B. T. West, R. J. A. Little, P. S. Boonstra, F. Alvarado-Leiton, Indices of non-ignorable selection bias for proportions estimated from non-probability samples. J. R. Stat. Soc. Ser. C. Appl. Stat. 68, 1465–1483 (2019). 10.1111/rssc.12371 PMC7724611 33304001

[51] R. J. A. Little, B. T. West, P. S. Boonstra, J. Hu, Measures of the degree of departure from ignorable sample selection. J. Surv. Stat. Methodol. 8, 932–964 (2020). 33381610 10.1093/jssam/smz023 PMC7750890

[52] P. S. Boonstra, R. J. A. Little, B. T. West, R. R. Andridge, F. Alvarado-Leiton, A simulation study of diagnostics for selection bias. J. Off. Stat. 37, 751–769 (2021). 34566235 10.2478/jos-2021-0033 PMC8460089

[53] A. Reinhart, R. Tibshirani, Big data, big problems: Responding to “are we there yet?”. arXiv:2109.00680 [stat.AP] (2021).

[54] W. Dempsey, Addressing selection bias and measurement error in COVID-19 case count data using auxiliary information. Ann. Appl. Stat. 17, 2903–2923 (2020). 10.1214/23-aoas1744 PMC11210953 38939875

[55] J. Fan, Y. Li, K. Stewart, A. R. Kommareddy, A. Garcia, J. O’Brien, A. Bradford, X. Deng, S. Chiu, F. Kreuter, N. Barkay, A. Bilinski, B. Kim, T. Galili, D. Haimovich, S. LaRocca, S. Presser, K. Morris, J. A. Salomon, E. A. Stuart, R. Tibshirani, T. A. Barash, C. Cobb, A. Gros, A. Isa, A. Kaess, F. Karim, R. Eliat, O. E. Kedosha, S. Matskel, R. Melamed, A. Patankar, I. Rutenberg, T. Salmona, D. Vannette, The University of Maryland Social Data Science Center Global COVID-19 Trends and Impact Survey, in partnership with Facebook (2020); https://covidmap.umd.edu/api.html.

[56] A. Reinhart, L. Brooks, M. Jahja, A. Rumack, J. Tang, S. Agrawal, W. al Saeed, T. Arnold, A. Basu, J. Bien, Á. A. Cabrera, A. Chin, E. J. Chua, B. Clark, S. Colquhoun, N. DeFries, D. C. Farrow, J. Forlizzi, J. Grabman, S. Gratzl, A. Green, G. Haff, R. Han, K. Harwood, A. J. Hu, R. Hyde, S. Hyun, A. Joshi, J. Kim, A. Kuznetsov, W. la Motte-Kerr, Y. J. Lee, K. Lee, Z. C. Lipton, M. X. Liu, L. Mackey, K. Mazaitis, D. J. McDonald, P. McGuinness, B. Narasimhan, M. P. O’Brien, N. L. Oliveira, P. Patil, A. Perer, C. A. Politsch, S. Rajanala, D. Rucker, C. Scott, N. H. Shah, V. Shankar, J. Sharpnack, D. Shemetov, N. Simon, B. Y. Smith, V. Srivastava, S. Tan, R. Tibshirani, E. Tuzhilina, A. K. van Nortwick, V. Ventura, L. Wasserman, B. Weaver, J. C. Weiss, S. Whitman, K. Williams, R. Rosenfeld, R. J. Tibshirani, An open repository of real-time COVID-19 indicators. Proc. Natl. Acad. Sci. U.S.A. 118, e2111452118 (2021). 34903654 10.1073/pnas.2111452118 PMC8713778

[57] Team CVoter, Cvoter news services (2020); https://cvoterindia.com/trackers/.

[58] The Times of India, Covid vaccination: Children between 15–18 years can register on CoWIN from Jan 1, on site from Jan 3 (2021); https://timesofindia.indiatimes.com/india/covidvaccination-children-between-15-18-years-can-register-on-cowin-from-jan-1-on-site-from-jan-3/articleshow/88531760.cms.

[59] Data for Good, Facebook, User guide for CTIS weights (2021); https://dataforgood.facebook.com/dfg/resources/user-guide-for-ctis-weights.

[60] D. Firth, K. E. Bennett, Robust models in probability sampling. J. R. Stat. Soc. Ser. B Stat. Methodol. 60, 3–21 (1998).

[61] J. Robins, M. Sued, Q. Lei-Gomez, A. Rotnitzky, Comment: Performance of double-robust estimators when “inverse probability” weights are highly variable. Stat. Sci. 22, 544–559 (2007).

