# Supplementary material for: Exploring the Big Data Paradox for various estimands using vaccination data from the global COVID-19 Trends and Impact Survey (CTIS)
Source: Sci Adv. 2024 May 31;10(22):eadj0266. doi: 10.1126/sciadv.adj0266 (PMC11314312; doi:10.1126/sciadv.adj0266)
Supplement: Supplementary file 1 — Sections S1 to S6 Figs. S1 to S3 Table S1 [file sciadv.adj0266_sm.pdf]

Science Advances
AAAS

Supplementary Materials for

Exploring the Big Data Paradox for various estimands using vaccination data from the global COVID-19 Trends and Impact Survey (CTIS)

Youqi Yang *et al.*

Corresponding author: Bhramar Mukherjee, bhramar@umich.edu

Sci. Adv. **10**, eadj0266 (2024)
DOI: 10.1126/sciadv.adj0266

This PDF file includes:

Sections S1 to S6
Figs. S1 to S3
Table S1

Supplementary texts

S1 Effective sample size of the successive difference in means

This section illustrates how to calculate the effective sample size of the successive difference in means, which is complementary to the section of Materials and Methods. We assume that the two simple random samplings are conducted at consecutive times with the same sample size and that the size of the finite population being studied N remains constant over time. Furthermore, we assume that the observations from the target surveys conducted at consecutive times are independent of each other. The variance of two SRS surveys can be computed by the following equation:

$$Var(\overline{Y}_{n_{eff},t} - \overline{Y}_{n_{eff},t-1}) = \frac{\sigma^2_{Y_{t-1}} + \sigma^2_{Y_t}}{N-1}\frac{N - n_{eff}}{n_{eff}}.$$

By equating the squared term of the estimation error $[(\bar{Y}_{n,t} - \bar{Y}_{n,t-1}) - (\bar{Y}_{N,t} - \bar{Y}_{N,t-1})]$ to the above variance, it yields the following equation:

$$\begin{aligned} n_{eff} &= \frac{(\sigma^2_{Y_{t-1}} + \sigma^2_{Y_t})\frac{N}{N-1}}{(\sigma^2_{Y_{t-1}} + \sigma^2_{Y_t})\frac{1}{N-1} + [(\overline{Y}_{n,t} - \overline{Y}_{n,t-1}) - (\overline{Y}_{N,t} - \overline{Y}_{N,t-1})]^2} \\ &\approx \frac{\sigma^2_{Y_{t-1}} + \sigma^2_{Y_t}}{[(\overline{Y}_{n,t} - \overline{Y}_{n,t-1}) - (\overline{Y}_{N,t} - \overline{Y}_{N,t-1})]^2}. \end{aligned}$$

S2 Effective sample size of the relative successive difference in means

This section illustrates how to calculate the effective sample size of the relative successive difference in means, which is complementary to the section of Materials and Methods. Similarly, we assume equal sample sizes in two SRS at consecutive times, unchanged population size, and independent observations. Using the Taylor polynomial of a function of two variables, the variance of two SRS surveys can be calculated by:

$$Var\Big(\frac{\overline{Y}_{n_{eff},t} - \overline{Y}_{n_{eff},t-1}}{\overline{Y}_{n_{eff},t-1}}\Big) = Var\Big(\frac{\overline{Y}_{n_{eff},t}}{\overline{Y}_{n_{eff},t-1}}\Big) \approx \frac{1}{n_{eff}}\Big(\frac{\overline{Y}_{N,t}}{\overline{Y}_{N,t-1}}\Big)^2\Big[\frac{\sigma^2_{Y_{t-1}}}{(\overline{Y}_{N,t-1})^2} + \frac{\sigma^2_{Y_t}}{(\overline{Y}_{N,t})^2}\Big].$$

By equating the squared term of the estimation error $[(\overline{Y}_{n,t} - \overline{Y}_{n,t-1})/\overline{Y}_{n,t-1} - (\overline{Y}_{N,t} - \overline{Y}_{N,t-1})/\overline{Y}_{N,t-1}]$ to the above variance, it yields the following formula:

$$n_{eff} = \frac{\left(\frac{\overline{Y}_{N,t}}{\overline{Y}_{N,t-1}}\right)^2 [\frac{\sigma^2_{Y_{t-1}}}{(\overline{Y}_{N,t-1})^2} + \frac{\sigma^2_{Y_t}}{(\overline{Y}_{N,t})^2}]}{[\frac{\overline{Y}_{n,t} - \overline{Y}_{n,t-1}}{\overline{Y}_{n,t-1}} - \frac{\overline{Y}_{N,t} - \overline{Y}_{N,t-1}}{\overline{Y}_{N,t-1}}]^2}.$$

S3 Reformulating the effective sample size of the successive difference in means

This section illustrates how to represent the effective sample size of the successive difference in means in terms of the three components of estimation error, which is complementary to the section of Materials and Methods. In equation [4], the denominator represents the squared term of the estimation error associated with the successive difference. By utilizing the decomposition of error across different dates, we can express the estimation error of the successive difference in the following manner.

$$(\overline{Y}_{n,t} - \overline{Y}_{n,t-1}) - (\overline{Y}_{N,t} - \overline{Y}_{N,t-1}) = (\overline{Y}_{n,t} - \overline{Y}_{N,t}) - (\overline{Y}_{n,t-1} - \overline{Y}_{N,t-1})$$
$$= \rho_{Y_t,R_t} \times \sqrt{\frac{N_t - n_t}{n_t}} \times \sigma_{Y_t} - \rho_{Y_{t-1},R_{t-1}} \times \sqrt{\frac{N_{t-1} - n_{t-1}}{n_{t-1}}} \times \sigma_{Y_{t-1}}.$$

S4 Reformulating the effective sample size of the relative successive difference in means

This section illustrates how to represent the effective sample size of the relative successive difference in means in terms of the three components of estimation error, which is complementary to the section of Materials and Methods. Using the second-degree Taylor polynomial of a function of two variables, we can express the estimation error of the relative successive difference in the following manner.

$$\frac{\overline{Y}_{n,t} - \overline{Y}_{n,t-1}}{\overline{Y}_{n,t-1}} - \frac{\overline{Y}_{N,t} - \overline{Y}_{N,t-1}}{\overline{Y}_{N,t-1}} = \frac{\overline{Y}_{N,t}}{\overline{Y}_{N,t-1}}$$

$$\times \left(\rho_{Y_t,R_t} \times \sqrt{\frac{N_t - n_t}{n_t}} \times \frac{\sigma_{Y_t}}{\overline{Y}_{N,t}} - \rho_{Y_{t-1},R_{t-1}} \times \sqrt{\frac{N_{t-1} - n_{t-1}}{n_{t-1}}} \times \frac{\sigma_{Y_{t-1}}}{\overline{Y}_{N,t-1}}\right)$$
$$\times \left(1 - \rho_{Y_{t-1},R_{t-1}} \times \sqrt{\frac{N_{t-1} - n_{t-1}}{n_{t-1}}} \times \frac{\sigma_{Y_{t-1}}}{\overline{Y}_{N,t-1}}\right).$$

S5 Variance for subgroup difference

This section presents the method for computing the value of σ^2_{Y*} for the error decomposition of the subgroup difference, which is complementary to the section of Materials and Methods. In our study of gender differences in vaccine uptake, both Y and G follow a Bernoulli distribution. By using the property of the variance under the Bernoulli distribution, we can derive,

$$Var(\sigma^2_{Y*}) = \overline{Y}_N(1 - \overline{Y}_N) + 4\overline{G}_N(1 - \overline{G}_N) + 4[E(Y = 1, G = 1) - \overline{Y}_N \times \overline{G}_N].$$

Here, $\overline{Y}_N$ is the population mean of the vaccination rate, $\overline{G}_N$ is the proportion of females among US adults, and E(Y = 1, G = 1) is the proportion of vaccinated females in the general adult population.

S6 Vaccine uptake in other countries

In addition to India and the US, we expanded our analysis of the estimation error of vaccine uptake from the CTIS to 85 other countries, using the formula by Meng (*9*). The benchmark data were sourced from the Johns Hopkins Coronavirus Resource Center (*12*). We developed an R Shiny app to showcase the results ((https://3ogdqc-youqi-yang.shinyapps.io/LLPinVaccine/). The R Shiny app offers two major advantages for future researchers. First, it extends the study period to include data from any available time in 2021, allowing the examination of temporary trends in estimation errors and their components. For instance, we observed a rapid increase in the inherent problem difficulty during the early stages of the national vaccine program, particularly in India between May and August 2021. Second, the app facilitates the exploration

of the Big Data Paradox in countries beyond India and the US. Notably, developed countries in Europe demonstrated lower estimation errors, lower ddc values, and larger effective sample sizes over time compared to low- and middle-income countries, especially those in Africa. This suggests that the selection bias in developed countries was less dominant. Across various countries, a noticeable disparity between the CTIS estimates and the benchmark data underscores that observations on the Big Data Paradox are not limited to specific geographic locations.

Supplementary figures

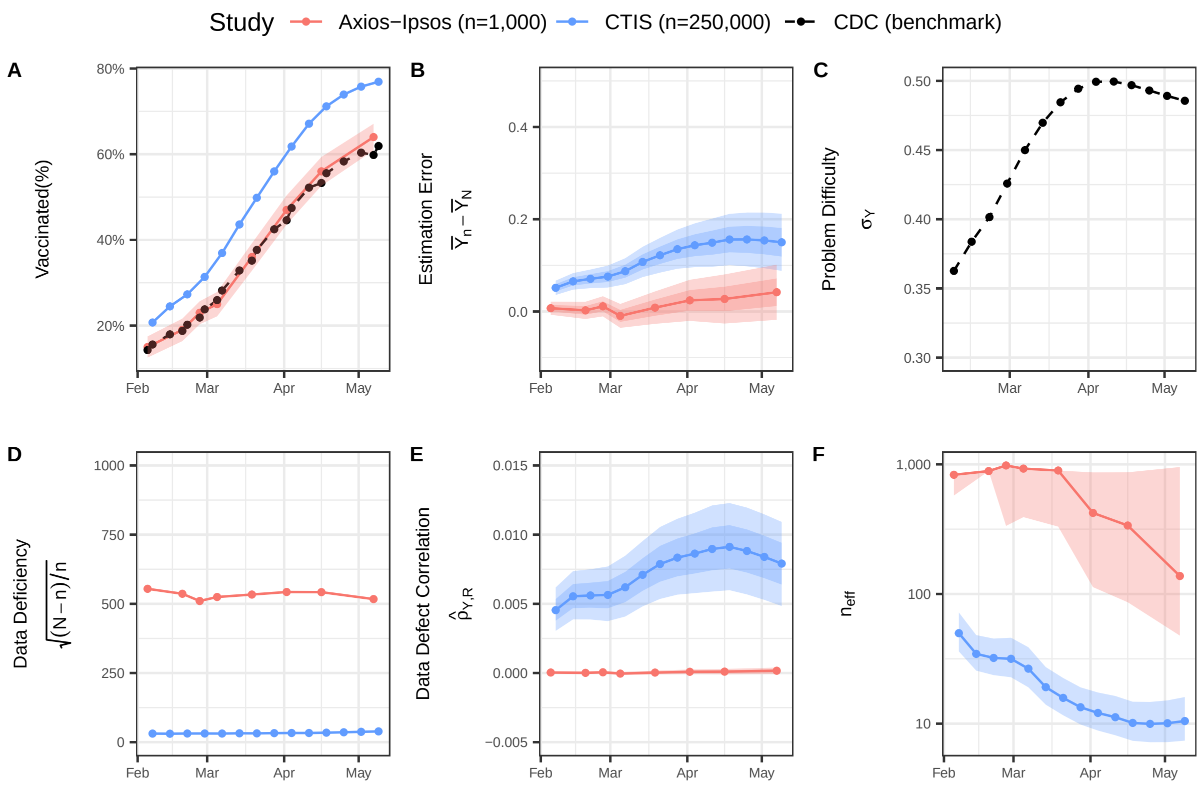

fig. S1. Decomposition of the estimation error and the effective sample size of vaccine uptake in the US. (A) The estimate, (B) estimation error, (C) inherent problem difficulty, (D) data deficiency, (E) data defect correlation, and (F) effective sample size of vaccine uptake among US adults from the CTIS (blue) and the Axios-Ipsos survey (red), compared to the CDC benchmark (black) between February 7 and May 15, 2021. For plot (A), shaded bands show the classic 95% confidence interval. For plots (B) and (E), shaded bands show the $\pm$ 5% and $\pm$ 10% benchmark imprecision adjustments. For plot (F), shaded bands show the $\pm$ 5% benchmark imprecision adjustments.

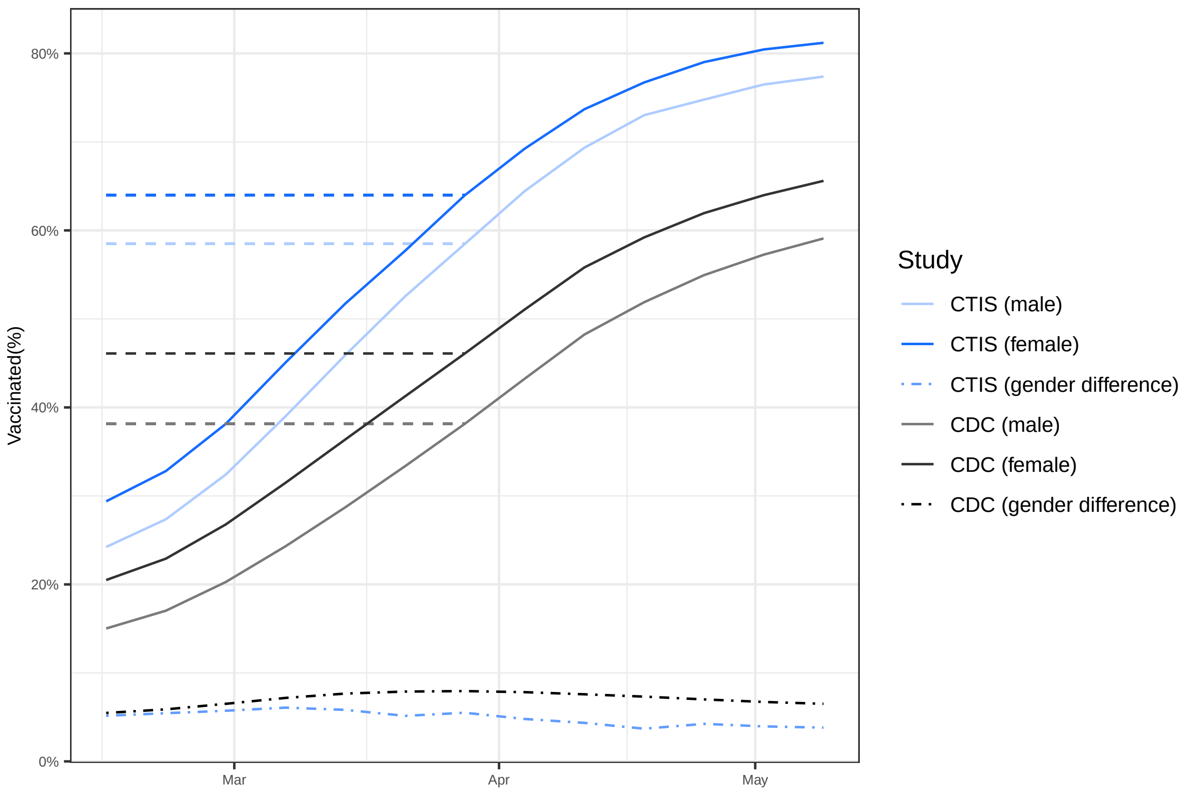

fig. S2. The difference in vaccine uptake between genders in the US. Estimates from the CTIS on vaccine uptake among US adults in males (light blue, solid), females (dark blue, solid), and the gender difference (blue, dot-dash) compared to the CDC benchmark (light gray, solid; dark gray, solid; black, dot-dash) between February 7 and May 15, 2021. The horizontal dashed lines indicate the median vaccination rate from each data regarding each gender.

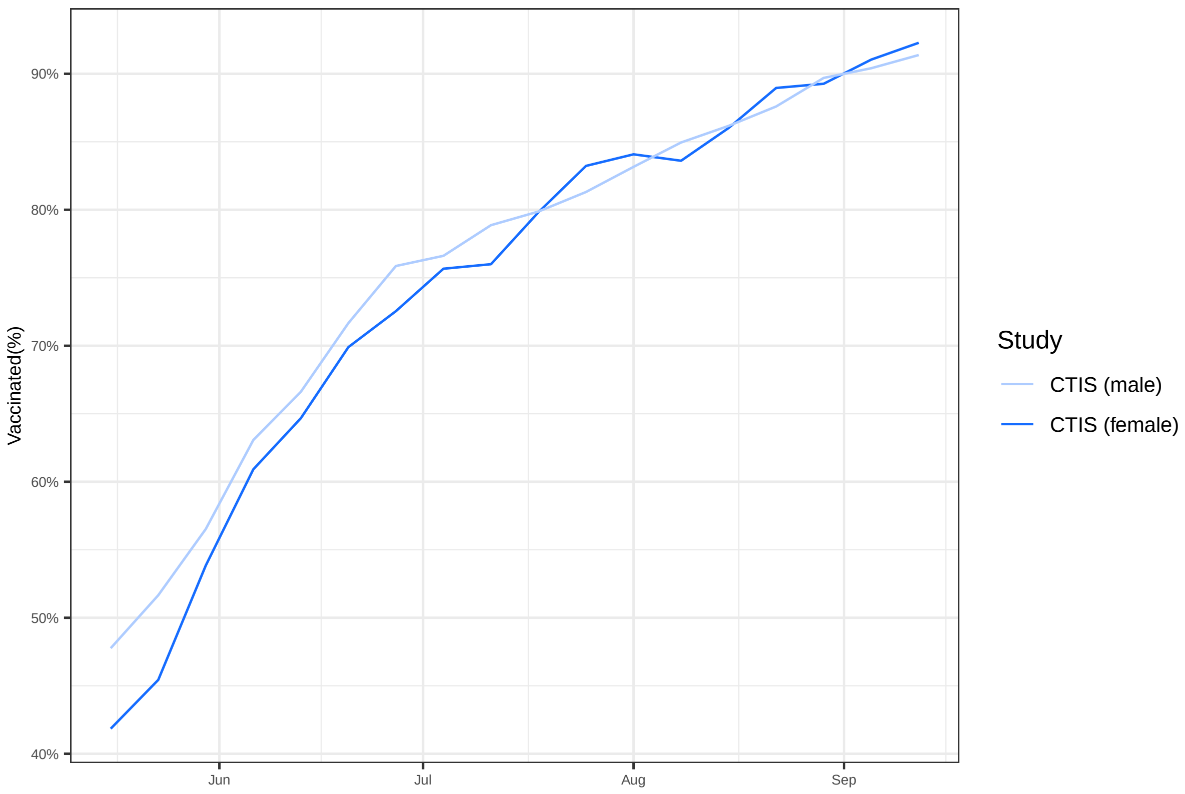

fig. S3. The difference in vaccine uptake between genders in India. Estimates from the CTIS on vaccine uptake among Indian adults in males (light blue, solid) and females (dark blue, solid) between May 16 and September 18, 2021.

Supplementary table

	US	
	**CTIS**	**Axios-Ipsos survey**
**Sample type**	Non-probability	Probability
**Target population**	US adults	US adults
**Sampling frame**	Adult Facebook Active User Base	Ipsos KnowledgePanel
**Recruitment mode**	Facebook newsfeed	Addressed-based mail sample
**Interview mode**	Online	Online
**Average sample size per week**	250,000	1,000
**Response rate**	1%	50%
**Vaccine uptake question**	"Have you had a COVID-19 vaccination?"	"Do you personally know anyone who has already received the COVID-19 vaccine?"
**Vaccine uptake responses**	"Yes"	"Yes, I have received the vaccine"
**Vaccine hesitancy question**	"If a vaccine to prevent COVID-19 were offered to you today, would you choose to get vaccinated?"	"How likely, if at all, are you to get the first generation COVID-19 vaccine, as soon as it's available"
**Vaccine hesitancy responses**	"No, probably/definitely not"	"Not very/at all likely"
**Weighting variables**	Age and gender	Age, gender, education, race/ethnicity, Census region, metropolitan status, income, and partisanship
**Sources for demographic benchmarks**	March 2017 CPS Supplement	March 2019 CPS Supplement

Table S1. Comparisons of the survey designs in the US. The benchmark data for vaccine uptake were sourced from the CDC reports.
